# Temperature orthogonal dynamic polymer networks

**DOI:** 10.1039/d5sc10098d

**Published:** 2026-03-04

**Authors:** Matthias Udo Mayer-Kriehuber, Evelyn Sattler, David Reisinger, Daniel Bautista-Anguís, Szymon Gaca, Pia Maria Egger, Fleana A. Sabatino, Sebastian Maar, Sandra Schlögl

**Affiliations:** a Polymer Competence Center Leoben GmbH Sauraugasse 1 A-8700 Leoben Austria sandra.schloegl@pccl.at sandra.schloegl@unileoben.ac.at; b Department of Polymer Engineering and Science, Technical University of Leoben Otto Glöckel-Strasse 2 Leoben A-8700 Austria; c Institute of Chemistry and Technology of Materials, Graz University of Technology Stremayrgasse. 9 A-8010 Graz Austria

## Abstract

Latent catalysts have gained increased attention for balancing high creep resistance with rapid (re)processability in covalent adaptable polymer networks (CANs). Among the reported systems, thermolatent catalysts offer particular advantages, as their activation is independent of part geometry, optical transparency, or irradiation depth, making them highly attractive for bulk materials and additively manufactured components. Here, a systematic study of thermobase generators (TBGs) with distinct activation and deactivation temperatures is presented, and their impact on bond-exchange-controlled stress relaxation in dynamic thiol–ene photopolymers undergoing transesterification is quantitatively assessed. Cyanoacetate- and oxalate-based TBGs, releasing amine bases at well-separated temperature windows, are investigated to directly correlate catalyst (de)activation with macroscopic flow behavior. Based on their non-overlapping thermal profiles, a cyanoacetate-based TBG releasing *N*,*N*,*N*′,*N*′-tetramethylguanidine and an oxalate-based TBG releasing 1,5,7-triazabicyclo[4.4.0]dec-5-ene are combined within a single CAN to realize temperature-orthogonal catalysis. Stress relaxation measurements demonstrate that the two catalysts operate independently and enable reversible, multi-cycle switching between four distinct bond-exchange regimes using temperature alone. This concept allows decoupling material stability under service conditions from rapid flow during reshaping, repair, or welding, and provides a versatile platform for applications requiring programmable mechanical response, such as soft robotic actuators, switchable adhesives or (re)processable additively manufactured components. As a proof of concept, multi-reshapable objects are fabricated *via* digital light processing 3D printing.

## Abstract 2

Thermolatent base generators with defined (de)activation temperatures are applied in dynamic photopolymers. Temperature orthogonal control over bond‑exchange kinetics is demonstrated allowing creep resistance and (re)processability to be decoupled.

## Graphical abstract

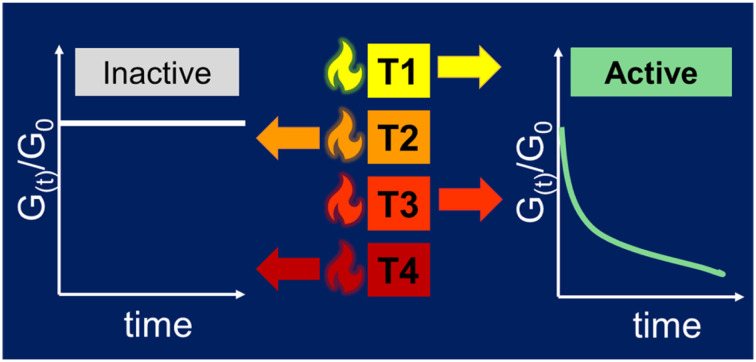

## Introduction

Dynamic polymer networks or covalent adaptable networks (CANs) are three-dimensional, covalently cross-linked polymers that combine the mechanical strength and thermal stability of thermosets with the reprocessability of thermoplastics.^1–5^ CANs contain dynamic covalent bonds that undergo reversible bond exchange, triggered by external stimuli (*e.g.*, heat, light or changing pH value), which leads to topological rearrangements in the network.^2,5,6^ In CANs following an associative bond exchange mechanism, the overall number of cross-links remains virtually constant, maintaining dimensional stability even at elevated temperatures and improving the material's resistance against creep and solvent stress cracking.^1–3,5,7,8^ Above the glass transition temperature (*T*_g_),^9^ the viscosity decreases in an Arrhenius-like manner, enabling reshaping, welding, and recycling of CANs.^5,10,11^ In 2010, Leibler *et al.* introduced the concept of thermo-activated CANs (which they termed vitrimers), by designing epoxy-acid and epoxy-anhydride networks that reshuffle bonds by catalysed transesterification between alcohol and ester groups.^3,7^ To date, the field of CANs is rapidly expanding and exploits various chemistries for bond exchange reactions such as transamination^1^ or transthioesterification.^12^ Despite significant advances, achieving precise spatiotemporal control over bond exchange reactions, and ensuring a good balance between high creep resistance and rapid bond exchange rates remains a challenge.^13^ In particular, creep resistance is a major limitation of CANs with a *T*_g_ below room temperature (RT). Once the *T*_g_ and the onset temperature for the bond exchange reactions are exceeded, the reshuffling of bonds and related viscous flow can lead to mechanical failure under constant load.^14^

This has sparked growing interest in the development of latent and switchable catalyst systems for regulating bond exchange kinetics.^15^ CANs relying on transesterification or thiol-thioester exchange reactions typically require the presence of catalysts to accelerate the bond exchange rate while lowering the onset temperature.^11,16^

By applying latent catalysts, the active species is released on demand in response to an external stimulus, whilst the material should exhibit good creep resistance as long as the catalyst remains in its not-activated state. For example, Bowman and co-workers employed a photobase generator (PBG), which released a strong guanidine base upon light exposure. In the presence of the liberated base, thiol–thioester exchange reactions were efficiently catalysed in a thiol–ene photopolymer. As a result, network rearrangement and material flow were observed even at RT.^17^ Our group extended this approach by developing a series of photolatent systems specifically suited for the use in dynamic polymer networks.^18^ In one approach, a quaternary ammonium salt (QAS) based on 1,5,7-triazabicyclo[4.4.0]dec-5-ene (TBD) was synthesised and incorporated into a soft thiol–epoxy network. Upon UV irradiation, the base was deliberately released facilitating viscoelastic flow at elevated temperatures.^19^ Building on this strategy, additional PBGs based on 1,5-diazabicyclo(4.3.0)non-5-ene (DBN) were synthesised and characterised.^20^ Parallel to this, we also explored photoacid generators in dynamic thiol-acrylate and thiol-thioester photopolymers.^21^ In a follow-up study, *N*,*N*,*N*′,*N*′-tetramethylguanidine (TMG) was employed as a building block in a QAS system. Release of the active base by means of visible light (405 nm) significantly increased the rate of bond exchange. Notably, this system exhibited a decrease in catalytic activity after prolonged exposure to temperatures exceeding 200 °C, which was related to the evaporation of free TMG. This observation led to the development of a CAN, exhibiting an irreversible dual control mechanism of bond exchange kinetics (light-induced activation and thermal deactivation).^22^ In a subsequent study, we demonstrated the reversible, light-driven spatiotemporal control of catalytic activity in a dynamic thiol–thioester network. Using a light-switchable base, catalyst activation was triggered by visible light and deactivation was obtained by UV light. Multiple ON/OFF cycles were achieved with only minor loss in catalytic efficiency.^23^ While such systems effectively demonstrate local control of the release of the catalysing species, they are limited by the optical transparency and geometry of the polymer samples.^24,25^ Thus, the uniform release of the catalyst across thick or filled materials remains difficult.^26^ To overcome these limitations, recent research efforts have been geared towards the development of thermolatent catalysts and CANs.^27^ Initially, we successfully synthesised a thermally activatable QAS based on TBD. The salt remained catalytically inactive during long-term storage at 50 °C but could be deliberately activated upon heating to 130 °C for 10 minutes. Through stress relaxation measurements, the activation and effectiveness of the system were confirmed in a dynamic thiol–ene polymer.^25^

Recently, we synthesised and characterised a comprehensive library of QASs with activation temperatures ranging from 60 to 290 °C.^28^ For cyanoacetate-based thermobase generators (TBGs), we systematically varied the base cation and demonstrated that both basicity and nucleophilicity influence thermal stability, with nucleophilicity having a more pronounced effect. By keeping the cation structure of the TBG constant, we further investigated the influence of various acetate and dicarboxylic acid-based anions. Here, the ability to form stabilised carbanions, rather than simple electron-withdrawing effects, was the key factor governing thermal stability.^28^ In a separate study, we expanded the library to include aromatic and multifunctional QASs, focusing on benzoate and citrate-based derivatives.^29^

Previous studies on thermally activated systems, including our own, primarily focused on one-time thermally activatable catalysts. However, industrial applications often require “multi ON/OFF” control, *e.g.*, for polymer parts, which are able to retain their high creep resistance after a repair step. Sardon *et al.* addressed this challenge by developing a TMG-based system that suppressed bond exchange below 100 °C but allowed reversible activation at higher temperatures.^30^ While effective, this approach relies on a thermal equilibrium and does not enable distinct switching states. As such, true ON/OFF switching a key feature for controlled reshaping or welding, remains unachievable with this method.

**Fig. 1:**
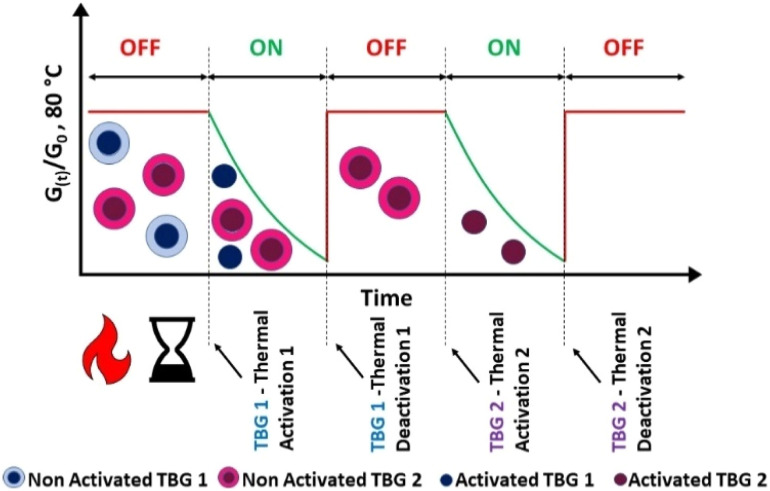
Schematic illustration of temperature orthogonal CANs containing two distinct activatable thermobase generators (TBG-1 and TBG-2). The normalised stress relaxation modulus ((*G*_(t)_/*G*_0_) at 80 °C) is plotted over time, showing sequential ON/OFF switching enabled by independent thermal activation (*T*_ACT_) and deactivation (*T*_DEACT_) of each TBG.

To overcome these limitations, we introduce the concept of temperature orthogonality, a strategy inspired by light-mediated wavelength-orthogonality principles previously demonstrated in dynamic networks.^31^ Unlike equilibrium-driven systems, temperature orthogonality employs multiple catalysts with non-overlapping activation and deactivation windows of temperature, allowing independent and sequential control of network dynamics. This sequential temperature orthogonality enables true ON/OFF switching of bond exchange reactions at distinct temperatures (Fig. 1).

## Results and discussion

### Influence of TBGs' cation structure on thermal (de)activation of bond exchange reactions in dynamic photopolymers

**Fig. 2:**
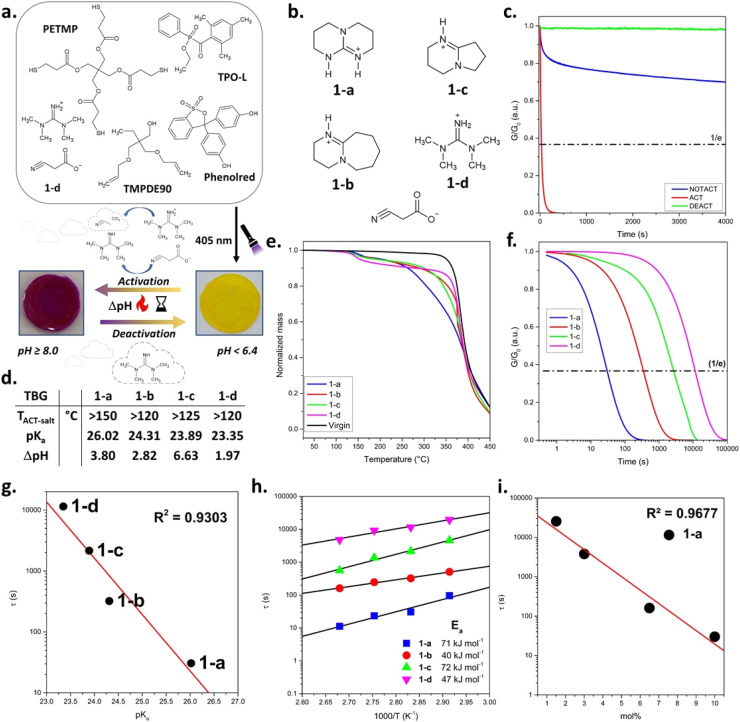
(a) Schematic illustration of the photocurable thiol–ene formulation and its thermally switchable activation using TBG 1-d. Upon light irradiation at 405 nm, a polymer network is formed. Thermal activation of 1-d liberates TMG and leads to a pH increase (ΔpH), as indicated by the phenol red colour change from yellow (pH < 6.4) to violet (pH ≥ 8.0).^34^ Upon thermal deactivation, involving the evaporation of TMG, the pH value decreases and the original yellow colour is restored. (b) Chemical structures of cyanoacetate-based QASs (TBG 1-a to 1-d). (c) Stress-relaxation measurements of thiol–ene based CANs containing 10 mol% 1-a. (d) Thermal behaviour of cyanoacetate-based TBGs (1-a to 1-d) with varying base strengths and structures. The activation temperatures in solid state (*T*_ACT-salt_), p*K*_a_ values of the TBG (in acetonitrile), and corresponding ΔpH values upon thermal activation are listed.^33^ (e) TGA curves of thiol–ene samples without TBG (virgin) and containing 10 mol% of 1-a to 1-d. (f) Normalised stress relaxation curves of dynamic thiol–ene networks containing different cyanoacetate-based TBGs (1-a to 1-d) measured at 80 °C. (g) Correlation between the literature p*K*_a_ values of the released bases and the nominal relaxation times τ* (*R*^2^ = 0.9303). (h) Arrhenius plots of nominal stress relaxation times (τ*) for TBGs 1-a to 1-d, showing the temperature dependence of relaxation and enabling the extraction of activation energies for each system and (i) plot of τ* *versus* TBG concentration (mol%) for compound 1-a, demonstrating a strong inverse correlation between catalyst concentration and relaxation time (*R*^2^ = 0.9677).

In the first series, cyanoacetate-based QASs bearing different base cations and thus, thermally releasing varying guanidine bases (1-a–d, Fig. 2b), were applied. Cyanoacetic acid (CA) was selected as stabilising anion as it decarboxylates at temperatures above 110 °C by releasing the base, CO_2_ and acetonitrile.^25,32^ The related p*K*_a_ values (from literature), *T*_ACT-salt_ (activation temperature of salt in solid state) and ΔpH (pH shift between activated and not-activated state measured in DMSO/water solutions) are summarised in Fig. 2d.^28,33^

To investigate the influence of TBG structure on thermal (de)activation of bond exchange reactions, a thiol–ene photopolymer network undergoing transesterification was used as a model system (Fig. 2a). The QASs did not compromise on cure kinetics between the radical-mediated reaction of pentaerythritol tetrakis(3-mercaptopropionate) across trimethylolpropane diallyl ether, and FTIR measurements confirmed full monomer conversion by the disappearance of the thiol stretching vibration at ∼2570 cm^−1^ and the allyl ether C=C out-of-plane deformation band at ∼925 cm^−1^ (Fig. S12 and S13a, SI).

Herein, the activation of each TBG was initially indicated by the visible colour change of embedded phenol red, which appears yellow under neutral conditions (pH ≈ 6.4) and turns red at mild basic conditions (pH ≥ 8) (Fig. 2a).^34^

To evaluate the thermal properties of the TBGs in the photopolymer networks, as well as the corresponding cleavage/evaporation times, TGA, differential scanning calorimetry (DSC) and stress relaxation measurements were conducted.

The activation and deactivation temperatures determined by TGA (*T*_ACT-TGA_ and *T*_DEACT-TGA_) represent the temperatures at which the TBG undergoes its most rapid activation/deactivation, as evidenced by a distinct mass drop in the TGA curve and a distinct peak in the first derivative of the TGA curve relative to the catalyst-free reference (virgin) network (Fig. 2e and S9 in SI). For all the investigated TBGs (1-a–d), the onset temperature (corresponding to the threshold temperature below which the TBG in the network remains stable) exceeded 100 °C (Fig. 2e and S9 in SI). Thus, subsequent stress relaxation experiments were carried out at 80 °C to ensure adequate creep resistance, and 3% deformation, which was within the region of linear elasticity (Fig. S13b and c in SI).

Prior to stress relaxation measurements, the samples were treated (under nitrogen atmosphere) at defined temperatures and times to release the catalysing species. Starting at 145 °C for 15 min, time and temperature were varied in increments of 20 °C and 10/15 min, respectively. The related stress relaxation curves of different thermally pre-treated CANs containing 1-a–d are provided in Fig. 2c, f and S14–S21 in SI.

*T*_ACT_ and *t*_ACT_ vary for each TBG, defined as the temperature and time the sample was treated, resulting in the fastest stress relaxation during rheological measurements (Fig. 2f and Table 1). It was found that *T*_ACT_ increases with rising basicity^33^ of the liberated base. This trend can be attributed to stronger ionic interactions between the base and the cyanoacetate anion, which enhance the thermal stability of the salt and thus require more energy for cleavage.^29^

**Table 1:** Thermal activation and catalytic performance of all investigated TBGs in the dynamic thiol–ene networks used. Values were obtained from stress relaxation experiments in which the highest relaxation rate was observed. Activation temperatures (*T*_ACT_), activation times (*t*_ACT_), stress relaxation times at 80 °C (τ*), and deactivation temperatures (*T*_DEACT_) are summarized

TBG	Concentration [mol%]	*T*_ACT_ [°C]	*t*_ACT_ [min]	τ* [s]	*T*_DEACT_ [°C]
1-a	10	145	15	31	250
1-b	10	145	15	321	240
1-c	10	125	30	2254	190
1-d	10	125	15	11 448	190
1-a	3	145	15	3738	250
2-a	3	190	30	4580	250
2-b	3	180	30	75 626	250
2-c	3	170	30	72 696	250

When either the temperature remained below *T*_ACT_ or the exposure time was shorter than *t*_ACT_, slower stress relaxation was observed during rheological measurements, which can be explained by a lower number of catalysing species. On the other hand, a rapid (1-c and 1-d) or gradual loss (1-a and 1-b) of catalytic activity was observed immediately after activation, either upon prolonged exposure (*t* > *t*_ACT_) at *T*_ACT_ or at *T* > *T*_ACT_. This behaviour is attributed to the volatility of the released base, which likely evaporates or diffuses out of the polymer matrix during thermal treatment. This explanation is supported by earlier observations of base evaporation in solid state revealed by EGA-FTIR spectroscopy.^28,29^

At the same heating rate, activation temperatures obtained *via* stress relaxation measurements (*T*_ACT_) deviated by ±20 °C from TGA and DSC data (*T*_ACT-TGA_–*T*_ACT-DSC_) (Fig. S10 and S11 in SI) and they were consistently lower than in the solid crystalline state (*T*_ACT-SALT_) (Fig. 2d and e). To verify the influence of the matrix, a Kissinger analysis was performed *via* DSC for a thiol–ene sample containing 10 mol% of 1-a.^28,35^ Measurements were conducted at heating rates of 5, 15, 25, and 35 K min^−1^, and the small endothermic peak corresponding to the activation event was used for the analysis (Fig. S22 and S23 in SI). The varying activation temperature values for different heating rates are shown in Table S7 in SI. The related Kissinger plot yields an activation energy of 104 kJ mol^−1^ and a strong linear correlation (*R*^2^ = 0.9893) (Fig. S24 in SI). Notably, this value lies between the previously reported activation energies determined in the solid (52 kJ mol^−1^) and dissolved state (140 kJ mol^−1^).^29^ Thus, these findings support the conclusion that the polarity of the surrounding medium/matrix and the mobility of the compound might be factors that influence the activation behaviour of the TBG.

Stress relaxation curves (each measured at 80 °C) of networks containing 1-a–d after thermal activation at their respective *T*_ACT_ for *t*_ACT_ are provided in Fig. 2f. A clear trend in the nominal relaxation times (τ*) is observed, following the order: 1-a < 1-b < 1-c < 1-d (Table 1). τ* (*τ*_KWW_) is defined as the characteristic relaxation time obtained from fitting the stress relaxation curves with a Kohlrausch–Williams–Watts (KWW) stretched exponential function *G*(*t*)/*G*_0_ = exp[−(*t*/*τ*_KWW_)^*β*^].^36^ Notably, 1-a releasing TBD caused particularly fast stress relaxation, with a τ* value in the range of highly dynamic CANs (τ* ≪ 100 s), indicating a rapid bond exchange once activated.^37^ By plotting τ* values against literature-reported p*K*_a_ values (in acetonitrile)^33^ for the corresponding free bases (Fig. 2g), a strong correlation (*R*^2^ = 0.9303) was found. This indicates that basicity is a dominant factor controlling exchange kinetics. Slight deviations from the linear trend may arise from additional factors such as nucleophilicity and steric hindrance of the bases.^28^

Arrhenius plots based on the networks' characteristic τ* (between 70 and 100 °C) are provided in Fig. 2h, S14–S21 and Tables S3–S6 in SI. Linear regression allowed the extraction of activation energies (*E*_a_) associated with the bond exchange process. A comparison of the *E*_a_ data reveals that no single molecular parameter, such as basicity (TBD > DBU > DBN > TMG) or nucleophilicity (TBD > DBN > DBU > TMG), fully dictates the catalytic efficiency.^33,38^ Instead, the observed trend in *E*_a_ (TBD > DBN > TMG > DBU) is expected to be a combination of electronic properties and structural effects, particularly ring strain, rigidity, and steric accessibility, that govern the energy barrier for transesterification in the networks.

Next, the influence of catalyst concentration on bond exchange kinetics was investigated using 1-a as a model system. The catalyst was incorporated into the thiol–ene network at varying concentrations of 1.5, 3.0, 6.5 and 10 mol% (relative to total functional thiol groups during network formation). A clear monotonic correlation was observed between catalyst concentration and τ*, measured at 80 °C after thermal activation, using the optimised conditions for 1-a (*T*_ACT_ = 145 °C and *t*_ACT_ = 15 min) (Fig. 2i). Such behaviour is consistent with previous studies on transesterification-based CANs, which report a pronounced acceleration of stress relaxation upon increasing TBD concentration.^39^ When plotted on a semi-logarithmic scale, the data follow an approximately linear trend over the investigated concentration range, corresponding to an exponential decrease of τ* with increasing catalyst concentration. Similar trends have been reported for TBD-catalysed dynamic polymers.^40^ In the present case, the smooth monotonic concentration dependence indicates that the thermally released base is effectively available within the network after activation. These findings corroborate the robustness of our activation protocol and further demonstrate the effective catalytic function of 1-a within the dynamic photopolymer.

Along with the activation, the deactivation step and related changes in network dynamics were studied comprehensively. In contrast to the activation step, defining the onset temperature for deactivation is challenging, as some TBGs showed slight reduction in catalytic activity immediately after base release. To ensure comparability and to determine the deactivation window, the deactivation time (t_DEACT_) was kept constant at 10 min while the temperature was varied in 10 °C increments. The first temperature at which a significant reduction in stress relaxation was observed, relative to the behaviour of the corresponding not-activated sample, was defined as *T*_DEACT_ (Table 1). Fig. 2c and S14–S21 in SI show the normalised stress relaxation curves of networks containing 1-a–d deactivated at their *T*_DEACT_. Overall, the relaxation curves show a close resemblance to the stress relaxation curves of the not-activated samples, due to the absence of the catalysing species.

Network degradation was excluded as a cause of deactivation as the maximum observed *T*_DEACT_ was 250 °C (for 1-a), and thus well below the network's degradation temperature obtained in TGA experiments (Fig. 2e). In addition, FTIR spectra taken prior to and after thermal exposure of 1-a (10 mol%) (Fig. S25a in SI) did not show any significant changes (*e.g.*, induced by oxidation). Therefore, it is expected that deactivation mainly proceeds *via* physical processes, such as base evaporation or diffusion, rather than chemical degradation of the polymer network, which aligns well with our previous EGA results.^28^ However, it cannot be excluded that some of the released amine bases are also degrading under the applied conditions and lose their catalytic activity.

The deactivation temperatures are summarised in Table 1. 1-d (releasing TMG) exhibited the lowest *T*_DEACT_ of 190 °C, while 1-a (releasing TBD) showed the highest one of 250 °C, which is directly related to the varying volatility of the corresponding free bases. This pronounced difference in volatility makes TMG and TBD a promising combination for the envisaged multi-stage thermoswitching of bond exchange kinetics. However, pairing 1-d with 1-a (or the other studied cyanoacetate-based derivatives) was not feasible as *T*_ACT_ of 1-a, 1-b and 1-c was overlapping with *T*_DEACT_ of 1-d. Among the studied TBGs, 1-d was the most promising candidate to be used in a temperature orthogonal CAN as it is stable up to ∼100 °C with only minor stress relaxation measured at 80 °C (see SI, Fig. S20c and 1-d, not-activated state). Moreover, 1-d has a relatively low activation temperature (*T*_ACT_ = 125 °C) and exhibited reduced catalytic activity at 145 °C due to the high volatility of TMG. In addition, the released TMG is an efficient catalyst for transesterification and the network underwent rapid stress relaxation at 80 °C (τ* = 188 min) (Table 1).

### Influence of TBGs' anion structure on thermal (de)activation of bond exchange reactions in dynamic photopolymer

**Fig. 3:**
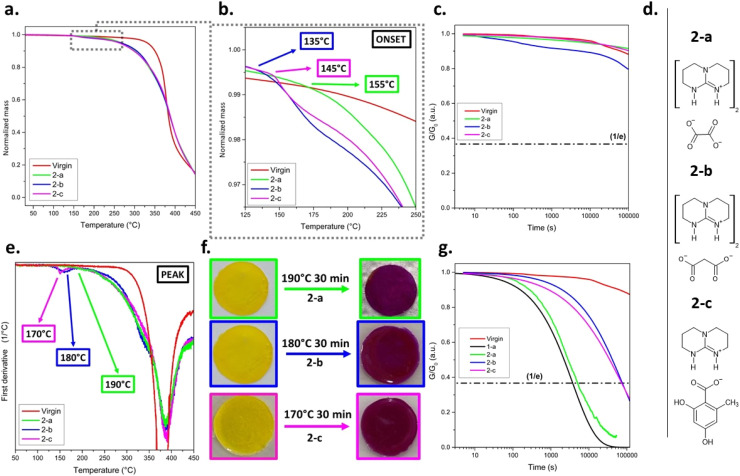
(a) Normalised TGA curves of virgin (catalyst-free) and CANs containing TBG 2-a, 2-b, and 2-c. (b) Magnified region of normalised TGA curves showing the temperature onset of mass loss of virgin and CANs containing TBG 2-a, 2-b, and 2-c. (c) Stress relaxation curves of the not activated CAN obtained at 80 °C. (d) Chemical structures of TBGs releasing TBD (2-a to 2-c). (e) First derivative of the TGA curves of virgin photopolymer and CANs containing TBG 2-a, 2-b, and 2-c. (f) Visual confirmation of activation through colour change of the embedded phenol red indicator after heating at the respective activation temperatures for 30 min. (g) Stress relaxation curves of not activated CAN obtained at 80 °C. The virgin network serves as a non-dynamic reference.

Due to the distinct difference in volatility between TMG and TBD, subsequent investigations focused on the performance of QSAs releasing TBD to be paired with TBG 1-d. Derivates were selected that showed a pronounced shift in pH (ΔpH > 2) and a thermal stability of > 140 °C in solution in previous studies.^28,29^ Based on these criteria, three different counteranions were selected: fully neutralized oxalate (2-a), malonate (2-b) and orsellinate (2-c, Fig. 3d and S25b in SI).

TGA revealed the following trend for the onset temperature of mass loss: 2-a > 2-c > 2-b, indicating increasing thermal stability in that order (Fig. 3a and b). *T*_ACT-TGA_ followed a slightly different trend: 2-a > 2-b > 2-c (Fig. 3e), reflecting differences in the efficiency and sharpness of thermal cleavage for each TBG. Although 2-b displayed a slightly lower onset activation temperature (135 °C) than the ideal threshold of 140 °C, it was still included for further evaluation.

Notably, TBG-containing networks (2-a–c) are less stable than the virgin reference network (Fig. 3a and b), and exhibited pronounced mass loss above 255 °C. This degradation is likely due to base-induced cleavage of ester bonds and limits the temperature window of the (de)activation processes.^41^

Subsequently, the time required to reach full activation (*t*_ACT_) at 190 °C was evaluated by stress relaxation measurements. All three formulations showed negligible stress relaxation in their not-activated state, with curves closely matching those of the catalyst-free (virgin) network (Fig. 3c and g). Subsequently, the networks were treated in 15 min intervals at 190 °C (*i.e.*, after 15, 30, and 45 min) and the related stress relaxation experiments (80 °C) revealed a clear trend in the catalytic performance: 2-a (τ* = 4580 s) ≪ 2-c (τ* = 72 696 s) < 2-b (τ* = 75 626 s) (see Table 1). All stress relaxation testing data were also consistent with the observed colour changes of the indicator dye (Fig. 3f, S27a and S35 in SI).

Whilst all three TBGs release TBD, the varying cleavage mechanisms strongly affect the catalytic activity of the liberated base. The network containing 2-a showed the fastest stress-relaxation behaviour which is related to the absence of reactive by-products and a favourable base release stoichiometry. 2-a undergoes a clean thermal cleavage yielding two equivalents of TBD per TBG molecule, with CO_2_ and potentially water and CO as the only by-products.

Although CO_2_ is formed during the cleavage process, the decomposition of 2-a appears to occur *via* low-molecular-weight fragmentation rather than decarboxylation, which requires the formation of a stable carbenium-ion, further minimizing interference with the exchange mechanism.^28^

These features resemble the cyanoacetate-based systems discussed previously, where CO_2_ and acetonitrile were released as inert by-products. To enable a comparison, additional samples containing 3 mol% of 1-a as a catalyst were prepared and evaluated under identical conditions (*T*_ACT_ = 145 °C and *t*_ACT_ = 15 min). When compared to 2-a, only minor differences in catalytic activity were observed, further supporting the assumption that both TBGs undergo efficient and clean activation.

In contrast, 2-c, despite being based on orsellinic acid, which is known to undergo decarboxylation, displayed significantly slower stress relaxation kinetics. Although the activation of orsellinate-based 2-c also proceeds *via* decarboxylation, a significantly slower stress relaxation rate (τ* was about 15 times higher than for 2-a) was observed.^42^ Partial activation can be ruled out, as full activation of 2-c was confirmed by TGA, visual colour change, and stress relaxation measurements as variations in either *T*_ACT_ or *t*_ACT_ did not lead to improved performance (Fig. 3 and S34b in SI). The poor catalytic efficiency is therefore likely attributable to the by-product orcinol, which possesses two acidic phenolic –OH groups and a high boiling point (∼291 °C).^29,42^

TBG 2-b performed similarly to 2-c, despite exhibiting a stronger ΔpH in solution-based assays.^28^ Like 2-c, 2-b showed an efficient activation behaviour based on colorimetric and TGA data, yet stress relaxation remained significantly slower than for 2-a (Fig. 3g and S34a in SI). Importantly, partial activation of 2-b cannot be ruled out. Prior work demonstrated that the activation of polyfunctional carboxylate-based TBGs can proceed stepwise and is highly medium-dependent.^28^ This could explain the reduced performance if only one TBD equivalent is released. Furthermore, the *β*-keto acid by-product formed during the thermal decomposition of 2-b may inhibit catalysis either *via* steric hindrance or chemical interaction with TBD.

To evaluate the influence of thermal cleavage by-products on the stress relaxation of CANs, an additional experiment was conducted to simulate the activation behaviour of the orsellinate-based 2-c.^29,43^ In particular, orcinol, the phenolic by-product generated upon thermal activation of 2-c, was co-formulated with cyanoacetate-based 1-a at stoichiometric molar ratios (1.5, 3.0, 6.5, and 10 mol%, relative to total functional thiol groups during network formation). All samples were thermally activated at 145 °C for 15 min (corresponding to *T*_ACT_ and *t*_ACT_ for 1-a), followed by stress relaxation experiments conducted at 80 °C. These data were compared with formulations containing only 1-a at identical concentrations. The results (Fig. S28b, c and Tables S8 and S9 in SI) reveal a gradual increase in τ* upon orcinol addition, with the difference becoming more pronounced at higher concentrations. At lower concentrations (1.5 mol%), the difference is less significant, but still observable. These findings suggest that although the underlying transesterification mechanism remains functional, the reaction kinetics are substantially suppressed by orcinol.

To further evaluate the inhibitory effect of orcinol on stress relaxation kinetics, Arrhenius plots were derived from samples containing 10 mol% of 1-a, with and without equimolar content of orcinol (Fig. S28b in SI). All samples were thermally activated at 145 °C for 15 min and stress relaxation measurements were conducted at 70, 80, 90, and 100 °C. The resulting Arrhenius plots showed good linearity for both data sets. The *E*_a_ without orcinol was calculated to be 75 kJ mol^−1^ (*R*^2^ = 0.9486), while the sample containing orcinol showed a slightly increased value of 89 kJ mol^−1^ (*R*^2^ = 0.8494). A clear and consistent shift to slower relaxation was observed across all temperatures corresponding to a constant increase in τ* by almost one order of magnitude (Δ*τ*_0_) (Fig. S28c in SI).

Overall, these results highlight the critical consideration in TBG design that by-products must not interfere with the catalyst function. Even when activation proceeds cleanly, by-products that remain within the polymer matrix and interact with the released base can significantly increase τ*.

Based on the comprehensive evaluation of thermal and catalytic properties, 2-a was selected as the second catalyst (TBG-2) for use in the multi-stage thermoswitchable CAN. Among the investigated derivatives, 2-a exhibited the highest thermal onset temperature (155 °C) and *T*_ACT_ (190 °C), ensuring that it remains inactive during the ON/OFF switching of TBG-1. Upon activation, 2-a demonstrated strong catalytic activity, reflected in the network's rapid stress relaxation behaviour, which was not affected by acidic by-products.

### Multiple temperature switchable CANs

Following the selection of TBG-1 (1-d) and TBG-2 (2-a), their combined incorporation into the polymeric network was investigated. A common limitation for QASs in such networks is their poor solubility, which is attributed to the ionic nature of the catalysts.^25^ At the same time, sufficient catalyst concentration is necessary to ensure effective stress relaxation after thermal activation. Therefore, TBG-1 (TMG-based) was incorporated at a concentration of 10 mol%, and TBG-2 (TBD-based) at 3 mol%. Incorporated separately at these concentrations, both TBGs showed a τ* below 15 000 s at 80 °C after thermal activation (Table 1 and Fig. S30 in SI). TBG-1 was activated at 125 °C for 15 min and deactivated at 145 °C to avoid any overlapping with the activation of TBG-2 carried out at 190 °C (Fig. 3e, g and S30a in SI). The deactivation time of TBG-1 had to be extended to 90 min to ensure efficient removal of the liberated TMG. Previous deactivation studies for TBG-1 were carried out at 190 °C for 10 min, but this temperature protocol would lead to undesired activation of TBG-2 (Fig. 3b, g, S27a and S30b in SI).

For simplicity, we refer to the activation step at 125 °C for 15 min as ACT1, and the deactivation at 145 °C for 90 min as DEACT1. To confirm the thermal stability of TBG-2 under these conditions, a control sample containing only TBG-2 was treated identically. No activation was observed, neither visually (*e.g.*, colour change) nor *via* stress relaxation measurements (Fig. S30b in SI). Furthermore, after undergoing the full treatment (TBG-1 activation followed by thermal deactivation), TBG-2 remained inactive, but could still be activated by a 30 min heat treatment at 190 °C. This confirms that the first (de)activation cycle did not impair the catalytic activity of TBG-2 (Fig. S30b in SI).

TBG-2 was then deactivated at 250 °C for 10 min (Fig. S30b in SI). In the following steps, we refer to the activation step at 190 °C for 15 min as ACT2, and the deactivation at 250 °C for 10 min as DEACT2. Unless otherwise stated, all treatments were applied sequentially in a fixed order: ACT1 → DEACT1 → ACT2 → DEACT2. Accordingly, any reference to a later step (*e.g.*, DEACT2) implies that all preceding steps were also performed in that order.

**Fig. 4:**
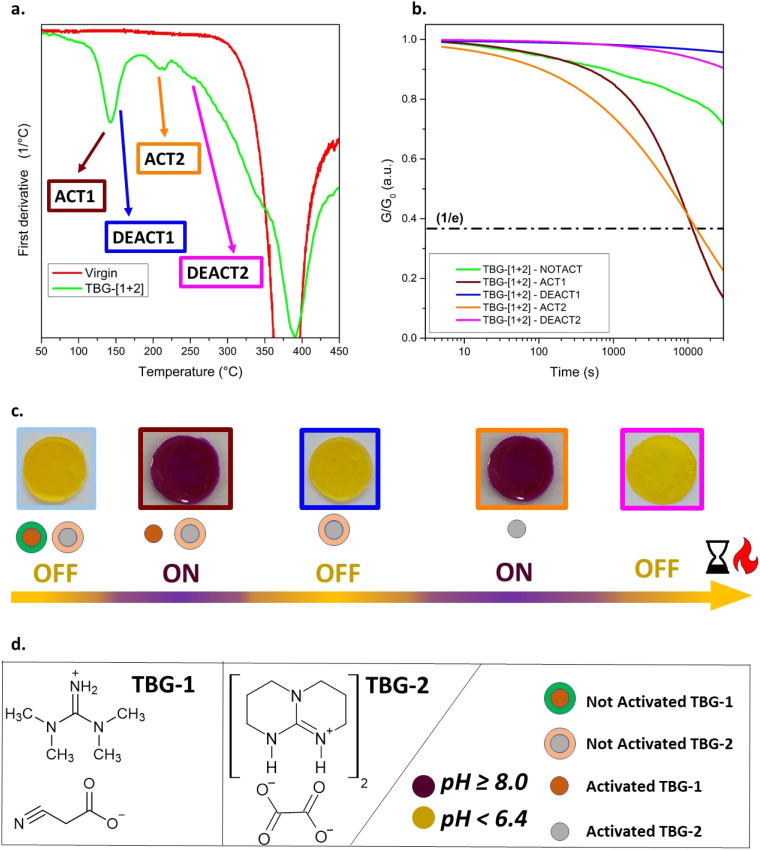
Multi-stage thermoswitching in a thiol–ene based CAN containing TBG-1 and TBG-2. (a) First derivative of TGA curves, comparing the TBG-containing sample with a catalyst-free network. The distinct activation (ACT1, ACT2) and deactivation (DEACT1, DEACT2) temperatures for TBG-1 and TBG-2 are marked. (b) Stress relaxation curves at 80 °C for different (de)activation states. (c) Visual colorimetric changes using phenol red to indicate base release and corresponding pH shifts for each (de)activation state. (d) Molecular structures of TBG-1 and TBG-2 and their corresponding colour changes: dark violet (pH ≥ 8.0, activated), yellow (pH < 6.4, deactivated).

In the following, the simultaneous implementation of TBG-1 and TBG-2 in the photopolymer was investigated. TGA was conducted on the prepared samples (Fig. S29a in SI), and first derivative of the obtained curves (Fig. 4a) revealed several distinct peaks corresponding to the characteristic (de)activation steps: between 120 and 160 °C (overlap of ACT1 and DEACT1), at 200 °C (ACT2), and at 255 °C (DEACT2). These transitions are consistent with DSC results (Fig. S29b in SI). A second activation cycle (ACT2) again induced the violet colouration, confirming that TBG-2 was activated independently of the first cycle. Importantly, this demonstrates that the first deactivation step did not originate from degradation of phenol red or the polymer network. Finally, after the last deactivation step (DEACT2), the colour shifted back to yellow, reflecting the complete evaporation of the remaining liberated base. Overall, these colorimetric changes visually confirm the temperature-dependent ON/OFF switching behaviour of TBG-[1+2] (Fig. 4c).

DSC measurements revealed only a minor shift in *T*_g_ from −16 °C for the virgin network to −13 °C for the dual-TBG system (Fig. S35a in SI). Comparing DSC curves of pure TBG salts (TBG-1 and TBG-2) with the corresponding photopolymer containing TBG-[1+2] revealed that the thermal event associated with TBG activation is shifted to slightly lower temperature in the polymer matrix (Fig. S35b and c).

Subsequently, stress relaxation measurements were performed on TBG-[1+2] after each (de)activation step (Fig. 4b). The not-activated (NOTACT) state already displayed slight stress relaxation, which is consistent with previous observations for TBG-1 (Fig. S30a in SI), and can be attributed to the residual basicity of the salt.^28^

Once thermally activated (ACT1), the system exhibited pronounced stress relaxation with τ* = 10 497 s, closely matching the τ* of the network containing TBG-1 alone (11 448 s; Fig. S30b in SI). This demonstrates that the incorporation of the second TBG did not compromise the performance of the first activation cycle. Following DEACT1, the catalytic activity was fully suppressed, and the relaxation behaviour matched that of the deactivated single-catalyst systems, confirming a complete and clean removal of the catalysing species (TMG). Notably, the stress relaxation curve showed even lower activity than the initial not-activated state, which is consistent with the lower basicity of TBD-based salts compared to TMG-based ones.^28,33^

In the second activation step (ACT2), the dynamic nature of the system was restored, and stress relaxation was observed again (Fig. 4b). However, τ* increased nearly three times compared to the single-catalyst TBG-2 system: τ* = 12 875 s for TBG-[1+2]versus τ* = 4580 s for TBG-2 alone. We attribute this effect to network rearrangements, which led to tighter networks and a lower number of –OH groups.^44^ Furthermore, upon activation, both TBG-1 and TBG-2 promoted accelerated stress relaxation, with ACT2 showing faster initial decay but a broader relaxation tail at longer times. Extended measurements up to 30 000 s revealed continuous relaxation without a clear plateau within the experimental window, although the relaxation rate decreased substantially at extended times.

Finally, the last deactivation step (DEACT2) resulted again in a suppression of catalytic activity, with no significant stress relaxation observed (Fig. 4b). This demonstrates that TBG-[1+2] possesses robust, sequential, and reversible ON/OFF switching capability across two activation/deactivation cycles.

### 3D printing of temperature switchable CANs

To demonstrate the practical applicability of multi-temperature switchable CANs, the formulation TBG-[1+2] was processed by digital light processing (DLP) 3D printing. The resin formulation exhibited a viscosity of 62 mPa s at a constant shear rate of 300 s^−1^ at room temperature (Fig. S31a in SI), which is favourable for the applied printing process. Rectangular samples with dimensions of 40.00 × 10.00 × 0.55 mm, containing phenol red, to observe ph-colour change upon thermal activation, were printed. Stress relaxation measurements confirmed that the printed samples retained the expected activation and deactivation behaviour across all four thermal treatment steps (ACT1, DEACT1, ACT2, DEACT2), consistent with non-printed, photocured control specimens (Fig. S31b in SI).

**Fig. 5:**
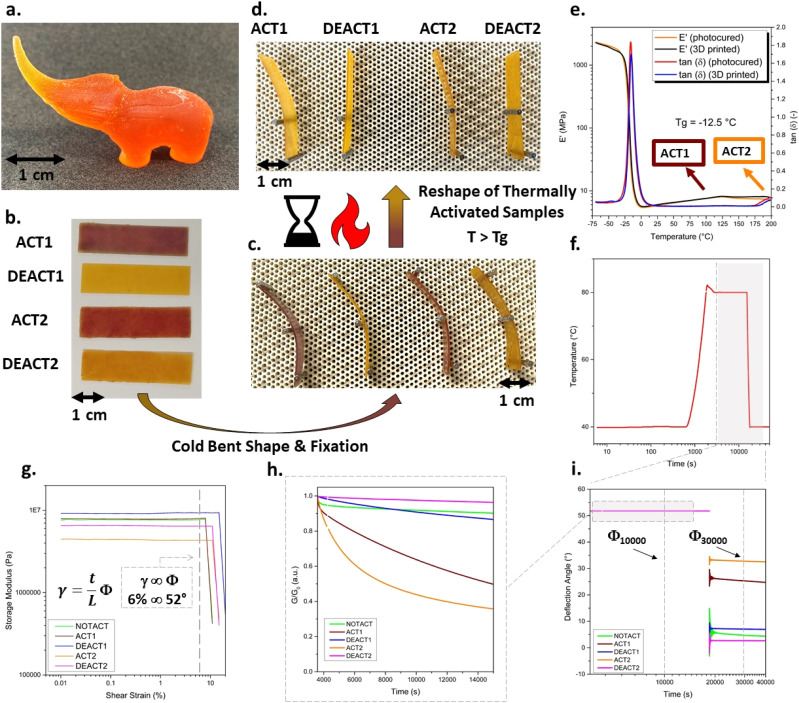
(a) Photograph of a 3D-printed elephant object. Repeated thermal reprogramming and shape fixation of TBG-[1+2] networks. (b) Rectangular 3D-printed samples of TBG-[1+2] were subjected to sequential thermal activation and deactivation steps (ACT1, DEACT1, ACT2, DEACT2). (c) Cold bending and mechanical fixation of all samples demonstrate their rigidity at room temperature. (d) Upon heating above the activation temperature of the respective TBGs, only the thermally activated samples (ACT1, ACT2) display shape reconfiguration, while the deactivated samples (DEACT1, DEACT2) retain their original bent shape. (e) DMA curve of the 3D-printed photopolymer containing TBG-[1+2]. Torsional reshaping experiments of photocured samples containing TBG-[1+2] (f) With applied temperature profile for samples in all five states (NOTACT, ACT1, DEACT1, ACT2, DEACT2) during experiments showing isothermal at 40 °C, heating to 80 °C, isothermal holding for stress relaxation, and subsequent cooling back to 40 °C prior to unloading and isothermal at 40 °C. (g) Strain amplitude sweep for samples in all five states (NOTACT, ACT1, DEACT1, ACT2, DEACT2), demonstrating linear viscoelastic behaviour up to ∼8–9% shear strain. (h) Stress relaxation at 80 °C under constant shear strain (*γ* = 6%) for approximately 3 h. (i) Retained deflection angle after cooling to 40 °C and unloading. Unactivated and deactivated samples recover elastically to <10% of the imposed deformation, whereas activated samples retain substantial permanent deformation (≈52% for ACT1 and ≈67% for ACT2 after 3 h).

Dynamic mechanical analysis (DMA) was conducted on 3D-printed TBG-[1+2] and storage modulus (*E*′) as a function of temperature is shown in Fig. 5e. All DMA measurements shown were performed on samples subjected to an additional 20 min UV post-curing step to ensure quantitative monomer conversion.

The material exhibits a glass transition temperature (*T*_g_) of −13 °C, consistent with DSC data (Fig. S35a in SI). Below *T*_g_, the network displays the expected high stiffness (*E*′ ≈ 10^3^ MPa), followed by a pronounced drop in modulus upon entering the rubbery regime. At elevated temperatures, two subtle but reproducible decreases in *E*′ are observed, with onsets near 100 °C and 180 °C. These events occur in close proximity to the activation temperatures of TBG-1 and TBG-2, and are expected to correlate with the release of the active base catalyst. Furthermore, DMA analysis revealed nearly identical storage modulus and tan *δ* profiles for photocured and 3D-printed specimens, confirming that the printing process does not significantly affect network formation or viscoelastic performance.

Next, rectangular 3D-printed samples containing TBG-[1+2] were thermally treated to sequentially reach the four defined states (ACT1, DEACT1, ACT2, DEACT2) (Fig. 5b). All samples were cold-bent and mechanically fixed at room temperature, demonstrating comparable rigidity and shape fixation under ambient conditions (Fig. 5c). After annealing at 80 °C for 20 h, the activated samples (ACT1 and ACT2) exhibited permanent shape reconfiguration (bent shape), whereas the deactivated samples (DEACT1 and DEACT2) retained their original geometry to a high degree and recovered elastically upon removal of the external constraint (Fig. 5d). These observations confirm the reversible and selective temperature orthogonality of the dual-TBG system over multiple reprogramming cycles.

To quantify shape programming and fixity, torsional deformation experiments were conducted using a rheometer operated in torsion mode.^11^ The applied temperature protocol is shown in Fig. 5f. Strain amplitude sweeps confirmed that a deformation amplitude of *γ* = 6% lies within the linear viscoelastic regime for all states (Fig. 5g). The *γ*–*Φ* conversion follows established torsion relations for rectangular specimens and was performed automatically by the rheometer software using the instrument geometry factors, with *Φ* obtained directly from *γ* (Fig. 5g).^11,45^ For the employed sample geometry, a shear strain of *γ* = 6%, corresponds to a deflection angle *Φ* = 52° (Fig. 5i). Under constant shear strain (*γ* = 6%, *Φ* = 52°) at 80 °C, activated samples (ACT1 and ACT2) exhibited pronounced stress relaxation, whereas unactivated and deactivated samples showed only minor modulus decay (Fig. 5h). After the thermal hold, samples were cooled and unloaded, and the retained deflection angle *Φ* was quantified (Fig. 5i and Table S10 in SI). Unactivated and deactivated samples recovered to below ∼10% (*Φ*_t_ = 30 000 s/*Φ*_t_ = 10 000 s) of the imposed deformation, while activated samples retained substantial permanent deformation (∼49% for ACT1 and ∼64% for ACT2 under the applied protocol). These results provide quantitative evidence that permanent shape reconfiguration occurs selectively in the activated state, while deactivated materials respond predominantly elastically and reversibly.

For the printing of more complex structures, phenol red was replaced with 0.05 wt% of the azo dye 1-(2,4-dimethylphenylazo)-2-naphthol (Sudan II). Sudan II acts as photoabsorber and to efficiently suppresses unwanted over-polymerisation. The change from phenol red to Sudan II led to a slight increase in viscosity to 100 mPa s (Fig. S33a in SI). With this formulation, a detailed elephant-shaped object was successfully printed (Fig. 5a).

## Conclusions

This work establishes thermolatent base generators (TBGs) as a robust and versatile strategy to achieve precise, temperature orthogonal control over bond-exchange kinetics in dynamic polymer networks. By systematically investigating the structure–property relationships governing TBG activation, deactivation, and catalytic efficiency, we identify the key molecular and environmental parameters that enable predictable ON/OFF switching of network dynamics. In thiol–ene based CANs, we demonstrate that both the cation (base) and the anion (acid) structure of TBGs critically determine their thermal activation/deactivation profile. Experiments with cyanoacetate-based TBGs releasing guanidine bases (TBD, DBU, DBN and TMG) show that the activation temperature (*T*_ACT_) scales with the basicity of the base cation, reflecting stronger ionic interactions within more stable salts. In contrast, the rate of stress relaxation after activation correlates strongly with the basicity of the liberated base, confirming that catalytic strength dominates bond-exchange kinetics. However, TBG activation is strongly matrix-dependent and affected by matrix polarity, steric confinement, and local mobility. Deactivation was shown to proceed predominantly *via* physical removal of the liberated base, governed by base volatility rather than network degradation or chemical side reactions. This insight enabled rational selection of complementary TBGs with non-overlapping (de)activation windows. In particular, pairing a cyanoacetate-based TBG (releasing volatile TMG) with a thermally more stable oxalate-based TBG (releasing TBD) enabled temperature orthogonal switching of the CAN yielding four reproducible ON/OFF states. This temperature orthogonal behaviour enables reversible access to multiple, independently addressable bond-exchange regimes within a single material, allowing material stability and processability to be decoupled through temperature alone rather than through changes in chemistry or composition. In contrast to classical associative dynamic networks, which typically require continuously elevated temperatures to enable bond exchange or exhibit undesired creep at service temperatures, the present TBG approach enables short thermal activation to trigger bond exchange while maintaining mechanical stability at lower temperatures. Quantitatively, stress relaxation times can be switched by more than one order of magnitude between activated and deactivated states, enabling rapid reshaping at elevated temperature while preserving dimensional stability under ambient conditions. Compared to photo-triggered latent catalysts, temperature orthogonal TBGs offer volumetric activation independent of optical penetration depth, fillers or irradiation geometry, enabling uniform activation in bulk materials and complex printed structures while avoiding light-induced gradients. Importantly, we demonstrate that cleavage by-products play a decisive role in the catalytic performance of the liberated base. Cleanly fragmenting systems such as cyanoacetate,– or oxalate-based TBGs, maintain high activity, whereas phenolic by-products can significantly suppress stress relaxation without fundamentally altering the exchange mechanism. This makes the compatibility of by-product a critical design criterion for the future development of TBGs. Beyond fundamental catalyst design, this insight is directly relevant for applications where repeated (de)activation cycles are required without accumulation of inhibitory species, such as recyclable adhesives, repairable coatings, or (re)processable soft active components. Finally, the temperature orthogonal TBG system was successfully processed by DLP 3D printing, retaining its full thermal switching behaviour in both simple and more complex geometries. Reshaping experiments confirmed that permanent shape reconfiguration occurs exclusively in the activated state, while deactivated materials respond elastically and reversibly to the outer stimulus. Beyond mechanistic understanding, temperature-orthogonal catalysis enables functionality that is difficult to achieve with single catalysts or photo-activated systems. The ability to reversibly decouple mechanical stability under service conditions from rapid bond exchange during reshaping, repair, or welding is particularly attractive for soft robotic components with temperature-tunable stiffness or damping, switchable adhesives allowing bonding and debonding without solvents or additives, and additively manufactured parts requiring post-printing reshaping or repair without loss of dimensional stability.

## Author contributions

M. U. Mayer-Kriehuber: conceptualization, investigation, methodology, validation, visualization, writing-original draft; E. Sattler: investigation, methodology; D. Reisinger: methodology, validation, writing – review & editing; D. Bautista-Anguís: investigation, validation, writing – review & editing; S. Gaca: investigation, writing – review & editing; P. M. Egger: investigation, writing – review & editing; F. A. Sabatino: investigation, writing – review & editing; S. Maar: investigation, methodology; S. Schlögl: funding acquisition, methodology, project management, supervision, validation, writing – review & editing.

## Conflicts of interest

There are no conflicts to declare.

## Supplementary Material

SC-OLF-D5SC10098D-s001

## Data Availability

The supporting data has been provided as part of the supplementary information (SI). Supplementary information: NMR, FTIR and UV-Vis spectra, TGA and DSC curves, stress relaxation data, viscosity results, Kissinger analysis and experimental section. See DOI: https://doi.org/10.1039/d5sc10098d.
