# Supplementary material for: Temperature orthogonal dynamic polymer networks
Source: Chem Sci. 2026 Mar 4;17(16):8260–71. doi: 10.1039/d5sc10098d (PMC12969069; doi:10.1039/d5sc10098d)
Supplement: SC-017-D5SC10098D-s001 [file SC-017-D5SC10098D-s001.pdf]

Supplementary Information (SI) for Chemical Science.
This journal is © The Royal Society of Chemistry 2026

Supporting Information

Temperature Orthogonal Dynamic Polymer Networks

M. U. Mayer-Kriehuber, E. Sattler, D. Reisinger, D. Bautista-Anguís, S. Gaca, P. M. Egger, F. A. Sabatino, S. Maar, and S. Schlögl*

M. U. Mayer-Kriehuber, E. Sattler, D. Reisinger, D. Bautista-Anguís, S. Gaca, P. M. Egger, F.A. Sabatino, S. Maar: Polymer Competence Center Leoben GmbH, Sauraugasse 1, A-8700 Leoben, Austria. E-Mail: sandra.schloegl@pccl.at

M. U. Mayer-Kriehuber, D. Reisinger, D. Bautista-Anguís, S. Gaca, F. A. Sabatino, S. Maar, S. Schlögl: Department of Polymer Engineering and Science, Technical University of Leoben, Otto Glöckel-Strasse 2, Leoben, A-8700, Austria.

P. M. Egger: Institute of Chemistry and Technology of Materials, Graz University of Technology, Stremayrgasse. 9, A-8010 Graz, Austria.

Table of Contents

Materials and Methods 2
Synthesis of thermobase generators (TBGs) 2
Characterisation methods 4
Sample preparation 6
Results 8
References 44

Materials and Methods

Materials

All chemicals were used as received without additional purification. 1,5,7-triazabicyclo(4.4.0)dec-5-ene (TBD), 1,8-diazabicyclo[5.4.0]undec-7-ene (DBU), 1,1,3,3-tetramethylguanidine (TMG), malonic acid (MA) and orsellinic acid (OA) were supplied by abcr GmbH (Karlsruhe, Germany). 1,5-diazabicyclo[4.3.0]non-5-ene (DBN), cyanoacetic acid (CA) and 1-(2,4-Dimethylphenylazo)-2-naphthol (Sudan II) were supplied by TCI Europe (Haven, Belgium). Methanol, diethylether and phenol red were purchased from Carl Roth (Karlsruhe, Germany). Oxalic acid (OX) was supplied by Sigma Aldrich (St. Louis, USA). Ethylphenyl(2,4,6-trimethylbenzoyl)phosphinate (TPO-L) and orcinol was purchased from BLD Pharmatech GmbH (Reinbek, Germany). Pentaerythritol-tetrakis(3-mercaptopropionat) (PETMP) trimethyl propane diallylether (TMPDE) were supplied from Bruno Bock (Marschacht, Deutschland).

Synthesis of thermobase generators (TBGs)

All syntheses were carried out according to the procedure described by Mayer-Kriehuber et al.in Ref [1].

Synthesis of 1-a to 1-d

All reactions were carried out under ambient atmosphere at room temperature (~22–25 °C). Cyanoacetic acid (CA) and the respective base (1.00 g) were each dissolved separately in 10 mL of methanol (MeOH) and stirred for 30 min to ensure complete dissolution. The base solution was then added dropwise to the CA solution under continuous stirring. The reaction mixture was stirred for 24 h at room temperature, and the reaction progress was monitored by TLC. After completion, the solvent was removed under reduced pressure. The crude product was purified by repeated washing with diethyl ether to afford the corresponding cyanoacetate salt. For all reactions, the amount of CA was adjusted to achieve a 1:1 molar ratio between acid and base. Compound-specific details, including the identity of the base, exact molar amounts, product name, isolated yield, physical form, and structural data, are reported below alongside the respective NMR spectra.

1-a — 1,5,7-Triazabicyclo[4.4.0]dec-5-enyl-2-cyanoacetate
Base used: 1,5,7-Triazabicyclo[4.4.0]dec-5-ene (TBD; 1.00 g, 7.18 mmol)
Yield: 93.5%
Physical appearance: White crystalline powder
^{1}H-NMR (DMSO-d_6, 600 MHz, δ): 9.43 (s,2H), 3.26 (t,4H), 3.18(s,2H), 3.16 (t,4H), 1.88 (m,4H) ppm. ^{13}C-NMR (DMSO-d_6, 600 MHz, δ): 166.62, 151.52, 118.91, 46.59, 37.71, 27.36, 20.85 ppm. FT-IR (cm^{-1}): 3220, 2940, 2250, 1610, 1520, 1350, 1320, 1200, 1050, 1015.

1-b — 1,8-Diazabicyclo[5.4.0]undec-7-enyl-2-cyanoacetate
Base used: 1,8-Diazabicyclo[5.4.0]undec-7-ene (DBU; 1.00 g, 6.57 mmol)
Yield: 94.6%
Physical appearance: White crystalline powder
^{1}H-NMR (DMSO-d_6, 600 MHz, δ): 10.71 (s,1H), 3.54 (t,2H), 3.47(t,4H), 3.25 (t,4H), 3.07 (s,2H), 2.70(m,2H), 1.90 (m,2H), 1.66 (m,2H), 1.61 (m,2H) ppm.
^{13}C-NMR (DMSO-d_6, 600 MHz, δ): 165.82, 164.29, 119.38, 53.71, 48.31, 38.03, 31.79, 28.77, 27.62, 26.49, 23.95, 19.43 ppm.

FT-IR (cm^{-1}): 3375, 3250, 3125, 2925, 2250, 1610, 1473, 1448, 1345, 1320, 1205, 1105, 980.

1-c — 1,5-Diazabicyclo[4.3.0]non-5-enyl-2-cyanoacetate
Base used: 1,5-Diazabicyclo[4.3.0]non-5-ene (DBN; 1.00 g, 8.05 mmol)
Yield: 91.2%
Physical appearance: Viscous yellowish resin
^{1}H-NMR (DMSO-d_6, 600 MHz, δ): 7.51 (s,1H), 3.54 (t,2H), 3.34 (t,2H), 3.27 (t,2H), 3.05 (s,2H), 2.75 (t,2H), 2.00 (m, 2H), 1.87 (m,2H) ppm.
^{13}C-NMR (DMSO-d_6, 600 MHz, δ): 163.66, 163.17, 119.01, 52.54, 41.99, 38.25, 29.71, 27.21, 18.58, 18.46 ppm.
FT-IR (cm^{-1}): 3380, 3270, 3220, 3060, 2945, 2885, 2250, 1615, 1345, 1305, 1265.

1-d — 1,1,3,3-Tetramethylguanidinyl-2-cyanoacetate
Base used: 1,1,3,3-Tetramethylguanidine (TMG; 1.00 g, 8.68 mmol)
Yield: 92.5%
Physical appearance: White crystalline powder
^{1}H-NMR (DMSO-d_6, 600 MHz, δ): 8.09 (s,2H), 2.93(s,2H), 2.89 (s,12H) ppm.
^{13}C-NMR (DMSO-d_6, 600 MHz, δ): 163.09, 161.13, 119.10, 39.30, 27.31 ppm.
FT-IR (cm^{-1}): 3340, 3140, 2960, 2250, 1600, 1565, 1410, 1345, 1260, 1100, 1065, 1035.

Synthesis of 2-a to 2-c

All reactions were performed under ambient atmosphere at room temperature (~22–25 °C). The respective carboxylic acid was dissolved in 10 mL of MeOH during stirring for 30 min. In a separate flask, TBD (1.00 g, 7.18 mmol) was dissolved in 10 mL of MeOH and stirred for 30 min. The TBD solution was then added dropwise to the acid solution under continuous stirring. The reaction mixture was stirred for 24 h at room temperature, and reaction progress was monitored by TLC. After completion, the solvent was removed under reduced pressure. The crude product was purified by repeated washing with diethyl ether to afford the corresponding TBD-carboxylate salt. For compound **2-c**, a monocarboxylic acid (OA; 7.18 mmol) was used with 1.00 g of TBD (7.18 mmol, 1.0 equiv.). For compounds **2-a** to **2-b**, dicarboxylic acids (3.59 mmol) were used with the same amount of TBD (1.00 g, 7.18 mmol), corresponding to 2.0 equivalents. As the reaction conditions were otherwise identical, specific details including the identity of the acid, product name, isolated yield, physical appearance, and structural data are provided below alongside the respective NMR spectra.

2-a - Bis(1,5,7-triazabicyclo[4.4.0]dec-5-enyl)-oxalate
Acid used: Oxalic acid (OX; 0.33 g, 3.59 mmol)
Yield: 95.6%
Physical appearance: White crystalline powder
^{1}H-NMR (DMSO-d_6, 600 MHz, δ): 10.30 (s,4H), 3.25 (t,8H), 3.13 (t,8H), 1.87 (m, 8H) ppm.
^{13}C-NMR (DMSO-d_6, 600 MHz, δ): 171.44, 151.04, 46.05, 37.16, 20.49 ppm.
FT-IR (cm^{-1}): 3385, 3270, 3205, 3150, 2975, 2885, 2190, 1625, 1570, 1440, 1365, 1325, 1295, 1200, 1070, 1020.

2-b - Bis(1,5,7-triazabicyclo[4.4.0]dec-5-enyl)-malonate
Acid used: Malonic acid (MA; 0.38 g, 3.59 mmol)

Yield: 92.7%
Physical appearance: White crystalline powder
^{1}H-NMR (DMSO-d_6, 600 MHz, δ): 10.63 (s,4H), 3.24 (t,8H), 3.12 (t,8H), 2.67 (s,2H), 1.86 (m, 8H) ppm.
^{13}C-NMR (DMSO-d_6, 600 MHz, δ): 174.46, 151.69, 46.56, 39.85, 37.60, 21.03 ppm.
FT-IR (cm^{-1}): 3300, 3220, 3155, 2965, 2880, 2760, 1955, 1710, 1640, 1560, 1440, 1360, 1320, 1300, 1225, 1205, 1160, 1068, 1030.

2-c - 1,5,7-triazabicyclo[4.4.0]dec-5-enyl-2,4-dihydroxy-6-methylbenzoate
Acid used: Orsellinic acid (OA; 1.21 g, 7.18 mmol)
Yield: 94.7%
Physical appearance: White crystalline powder
^{1}H-NMR (DMSO-d_6, 600 MHz, δ): 9.18 (s, 2H), 8.63 (s, 2H), 5.88 (s, 2H), 3.29 (t, 4H), 3.21 (t, 4H), 2.45 (s, 3H), 1.90 (m, 4H) ppm.
^{13}C-NMR (DMSO-d_6, 600 MHz, δ): 174.38, 165.87, 159.48, 151.49, 142.89, 110.67, 100.53, 46.62, 37.80, 23.98, 20.85 ppm.
FT-IR (cm^{-1}): 3305, 3217, 2961, 2877, 2542, 1638, 1564, 1456, 1358, 1290, 1243, 1199, 1159, 1108, 1054.

Characterisation methods

^{1}H and ^{13}C nuclear magnetic resonance (NMR) spectra were recorded on a Bruker Ascend 600 MHz spectrometer (Bruker, USA) with deuterated DMSO as solvent and trimethylsilane (TMS) as internal standard.
Fourier-transformation infrared (FTIR) spectroscopy was conducted between 500 and 4000 cm^{-1} with a FTIR Alpha II spectrometer (Bruker, USA). The device was equipped with a Bruker Platinum ATR unit for attenuated total reflection using a diamond crystal. All spectra were accumulated from 24 scans at a resolution of 4 cm^{-1}.
Thermal gravimetric analysis was carried out with a TGA/DSC3+ thermogravimetric analyser (Mettler Toledo, USA). All measurements were performed with a heating rate of 10°C min^{-1} in a temperature range from 25 °C to 900 °C under nitrogen flow of 50 mL min^{-1}. ~ 10 mg of samples were used for these experiments.
Differential scanning calorimetry measurements were performed with a DSC 3 instrument (Mettler Toledo, Switzerland) under nitrogen flow of 50 mL min^{-1}. ~ 10 mg of samples were used for these experiments.
Standard measurements DSC of thiol-ene samples were conducted with the following heating cycle: (1) hold for 1 min at 25 °C, (2) cooling from 25 °C to -50 °C with 10 °C min^{-1} (3) hold for 5 min at -50 °C, (4) heating from -50 °C to 270 °C with 10 °C min^{-1}, (5) hold for 1 min at 270 °C, (6) cooling from 270 °C to 25 °C with 10 °C min^{-1}.
DSC measurements for Kissinger analysis of thiol-ene samples were conducted at the heating/cooling rates of 5, 15, 25 and 35 k min^{-1}. Every measurement had the same cycle, with only changes in the heating/cooling rates: (1) hold for 1 min at -25 °C, (2) heating from -25 °C to 250 °C, (3) hold for 1 min at 250 °C, (4) cooling from 250 °C to -25 °C, (5) hold for 1 min at -25 °C, (6) heating from -25 °C to 250 °C, (7) hold for 1 min at 250 °C, (8) cooling from 250 °C to 25 °C.
DSC measurements of salts were conducted with the following heating cycle:

1 min at 25 °C, (2) cooling from 25 °C to -50 °C, (3) hold for 5 min at -50 °C, (4) heating from -50 °C to 295 °C with 10 °C min-1, (5) hold for 1 min at 295 °C, (6) cooling from 295 °C to 25 °C with 10 °C min-1. 10 mg of sample were used.

Dynamic mechanical analysis (DMA) was performed on a NETZSCH Proteus 90 DMA 303 instrument (Selb, Germany). Temperature-sweep measurements were carried out in a continuous mode from −70 °C to 200 °C with a heating rate of 2.0 K min^{-1}. A dynamic load frequency of 1.0 Hz was applied using a dynamic displacement of 0.01 mm and a static strain limit of 50%. The normal force was set to 0.02 N, with a contact force control range of 0.005 N and a proportional factor of 1.2.

UV–Vis absorption measurements were performed on a Varian Cary 50 UV–Visible spectrophotometer (Palo Alto, USA) in the range of 200–500 nm, using a scan rate of 600 nm min^{-1} and a data interval of 1.00 nm. A quartz cuvette with an optical path length of 10 mm was used. The absorption spectra of the TBGs were recorded at a concentration of 1×10^{-3} mol L^{-1} in acetonitrile.

Stress-relaxation experiments were performed on a MCR 702e rheometer (Anton Paar, Graz, Austria). Thiol–ene samples were punched from moulded plates using a 10 mm circular die. For each measurement series, specimens were derived from a single plate to ensure consistent conditions.

Amplitude sweep measurements were conducted using a parallel-plate geometry (10 mm diameter). The samples were equilibrated at 80 °C prior to measurement. Oscillatory shear was applied at a constant frequency of 1.0 Hz, while the strain amplitude was increased logarithmically from 0.01 % to 100 % over 30 measurement points. A constant normal force of 10 N was applied throughout the experiment using automated gap control. The amplitude sweep was used to identify the linear viscoelastic regime for subsequent stress relaxation measurements. For rectangular specimens used in torsional programming experiments (25 × 5 × 1 mm), amplitude sweeps were additionally performed in torsion mode under otherwise identical conditions (80 °C, 1.0 Hz, logarithmic strain sweep from 0.01 % to 100 %), using a constant tensile preload of −0.05 N to maintain sample alignment. In both geometries, amplitude sweeps were used to determine the linear viscoelastic regime.

Thiol–ene networks were tested using a parallel-plate geometry (10 mm diameter) in relaxation-after-shear mode. The samples were equilibrated at the target temperature for 10 min, after which a 3% step strain was applied. A constant normal force of 10 N was used, with automated gap control. Activated thiol–ene samples were measured from 70 to 110 °C in 10 °C intervals, whereas non-activated and deactivated samples were measured at 80 °C.

Viscosity of the uncured resin formulations was measured at 25 °C using a parallel-plate geometry with a diameter of 25 mm and a constant gap of 0.2 mm. The measurement protocol consisted of a linear increase in shear rate from 0 to 300 s^{-1} over 5 min, followed by a constant shear rate of 300 s^{-1}.

All reshape experiments were performed in a HeraTherm convection oven, Thermo Scientific (Waltham, USA).

Rectangular specimens (25 × 5 × 1 mm) were characterized in torsion mode using an Anton Paar MCR rheometer equipped with a solid rectangular clamp geometry. All measurements were performed under displacement control with a constant normal force of −0.05 N to maintain sample contact without introducing axial preload.

Samples were first equilibrated at 40 °C for 600 s at zero shear strain, followed by heating from 40 to 80 °C over 1200 s and subsequent equilibration at 80 °C for 1800 s. A shear strain of 6% was then applied and held at 80 °C for approximately 11,600 s to allow stress relaxation and network rearrangement. After the relaxation period, samples were cooled from 80 to 40 °C over 1200 s and equilibrated at 40 °C for 2100 s while maintaining the imposed strain. The shear strain was then released, and a minimal residual torque of 0.001 mN·m was applied to avoid instrument drift while

allowing free elastic recovery. The resulting deflection angle was monitored for up to 90 000 s at 40 °C.

This protocol was applied identically to samples in all five states (NOTACT, ACT1, DEACT1, ACT2, DEACT2). The retained deflection angle after unloading was used as a quantitative measure of permanent shape reconfiguration.

Thiol–ene specimens were produced by DLP 3D printing and post-cured under identical conditions. Rectangular samples with dimensions of 40 × 10 × 0.55 mm were used for reshaping experiments. Prior to reshaping, the specimens were subjected to defined thermal treatment steps corresponding to activation and deactivation sequences (ACT1, DEACT1, ACT2, DEACT2).

Following thermal conditioning, the samples were mechanically deformed into a cold-bent configuration and fixed in place using an external fixture. The fixed specimens were then placed in the convection oven at 80 °C for 20 h. After annealing, the external constraint was removed and the samples were cooled to room temperature.

In addition to rectangular samples, 3D-printed stickman-shaped specimens were processed analogously. These were likewise thermally conditioned (ACT1/DEACT1), mechanically deformed into a defined geometry, fixed in place, and annealed in the convection oven at 80 °C for 20 h before cooling.

Sample preparation

Thiol–ene resin formulations were prepared by mixing PETMP (4.91 mmol, 0.600 g), TMPDE-90 (4.91 mmol, 0.553 g), phenol red (0.045 wt%, 1.6×10^{-3} mmol, 0.565×10^{-3} g), and the respective TBGs in a 5-mL glass vial. TBG quantities were weighed according to **Table S01**. In selected experiments then varying amounts of Orcinol were added **Table S02**. The mixtures were stirred on a magnetic stirrer for 30 min until complete dissolution of the TBG. Subsequently, 1.15 mol% of the photoinitiator Irgacure® TPO-L (7.7×10^{-2} mmol, 0.018 g) was added, and the resin was stirred for an additional 10 min to ensure homogeneity.

For cast, the resin was poured into a prefabricated silicone mould and cured using a Lama lamp (Opsytec Dr. Gröbel, Ettlingen, Germany) operating at 405 nm. Curing was performed at a lamp–sample distance of 10 cm, at 30% intensity (24.00 mW cm^{-2}), for 20 s . After curing and demoulding, transparent polymer films with a thickness of 1 mm were obtained.

For preparation of rectangular specimens used in torsional deformation experiments, the same resin formulation was cast into a prefabricated silicone mould (≈10 cm diameter, 1 mm thickness). These samples were cured in a Form cure system (Formlabs, USA) with a 405 nm LED source for 60 min at room temperature. After curing, the resulting plates were manually cut into rectangular specimens (25 × 5 × 1 mm) using a scalpel prior to rheological testing.

For 3D-printed thiol–ene specimens, the same resin formulation was used without modification and was upscaled to a total batch size of 40 g. Printing was performed via digital light processing (DLP) using a prototype "Doppio" DLP printer (W2P Engineering GmbH, Austria), equipped with a 405-nm LED light engine from In-Vision Digital Imaging Optics GmbH. The system operated at a pixel resolution of 50 µm and a light intensity of 3 mW cm^{-2} with a single layer gap 100 um and irradiation time 60 s. For printing of more complex 3D geometries, an alternative formulation was prepared in which phenol red was replaced by Sudan II as the dye. Sudan II was used at 0.05 wt%, corresponding to 0.565×10^{-3} g per batch based on 0.600 g PETMP equivalent resin mass.

Table S1: Quantities of TBGs used for the thiol-ene CANs

TBG	mol%	mmol	g
**1-a**	10	0.49	0.110
**1-a**	6.5	0.32	0.072
**1-a**	3	0.15	0.033
**1-a**	1.5	0.07	0.017
**1-b**	10	0.49	0.117
**1-c**	10	0.49	0.103
**1-d (TBG-1)**	10	0.49	0.098
**2-a (TBG-2)**	3	0.15	0.054
**2-b**	3	0.15	0.056
**2-c**	3	0.15	0.045

Table S2: Quantities of additives used for the thiol-ene CANs

Additive	mol%	mmol	g
Orcinol	10	0.49	0.061
Orcinol	6.5	0.32	0.040
Orcinol	3	0.15	0.018
Orcinol	1.5	0.07	0.009

Thermal Activation and Deactivation: Thermal activation and deactivation procedures were carried out using a MR-HEITEC magnetic stirrer (Heidolph, Germany). The device allowed temperature control from room temperature up to 250 °C. All treatments were performed under a nitrogen atmosphere, using a continuous nitrogen flow of 50 mL min^{-1} to maintain an inert environment throughout the activation or deactivation process.

Results

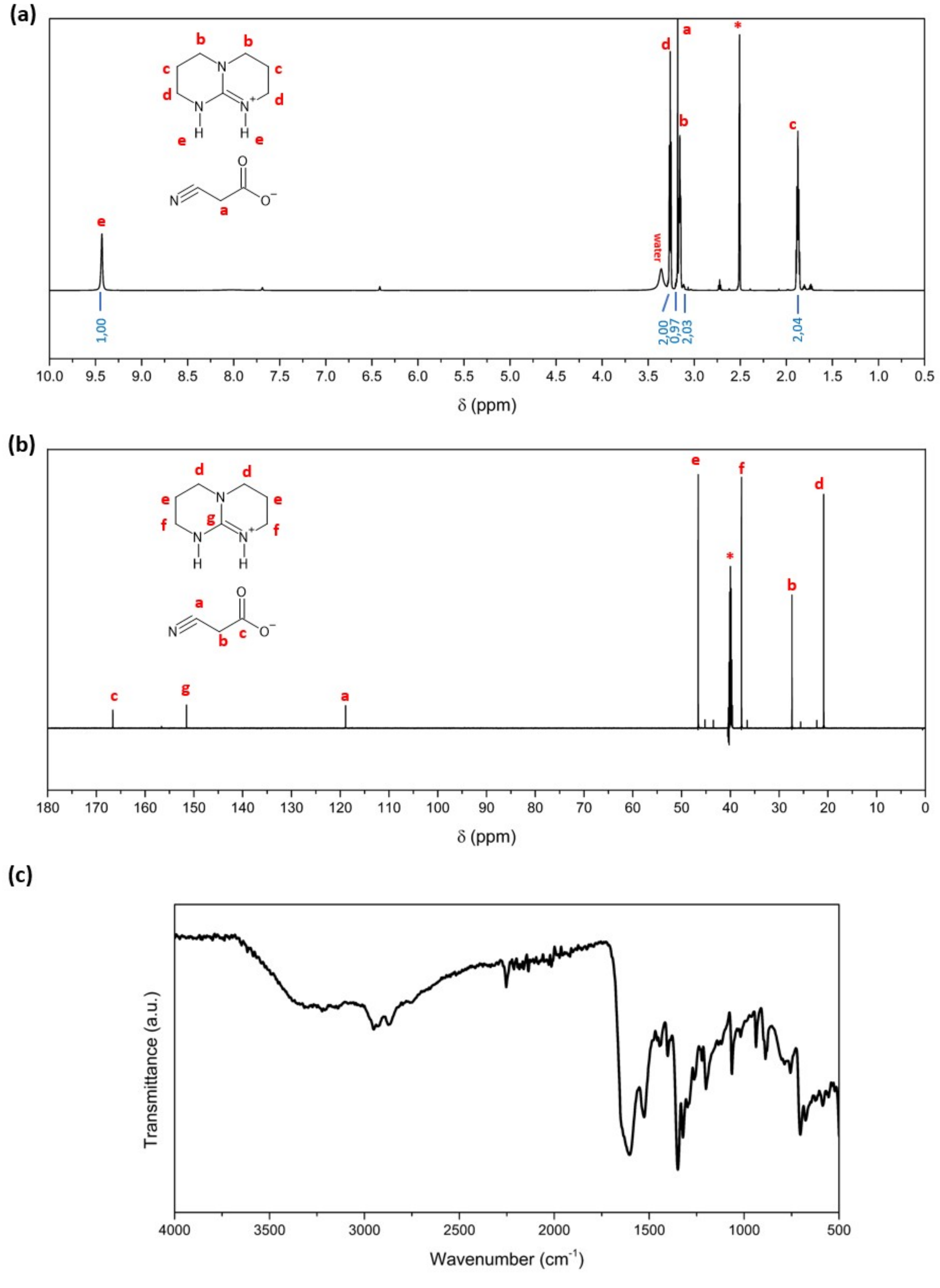

Figure S1: (a) ^{1}H NMR, (b)^{13}C NMR (600 MHz, DMSO -d_6), and (c) FTIR spectra of **1-a**.

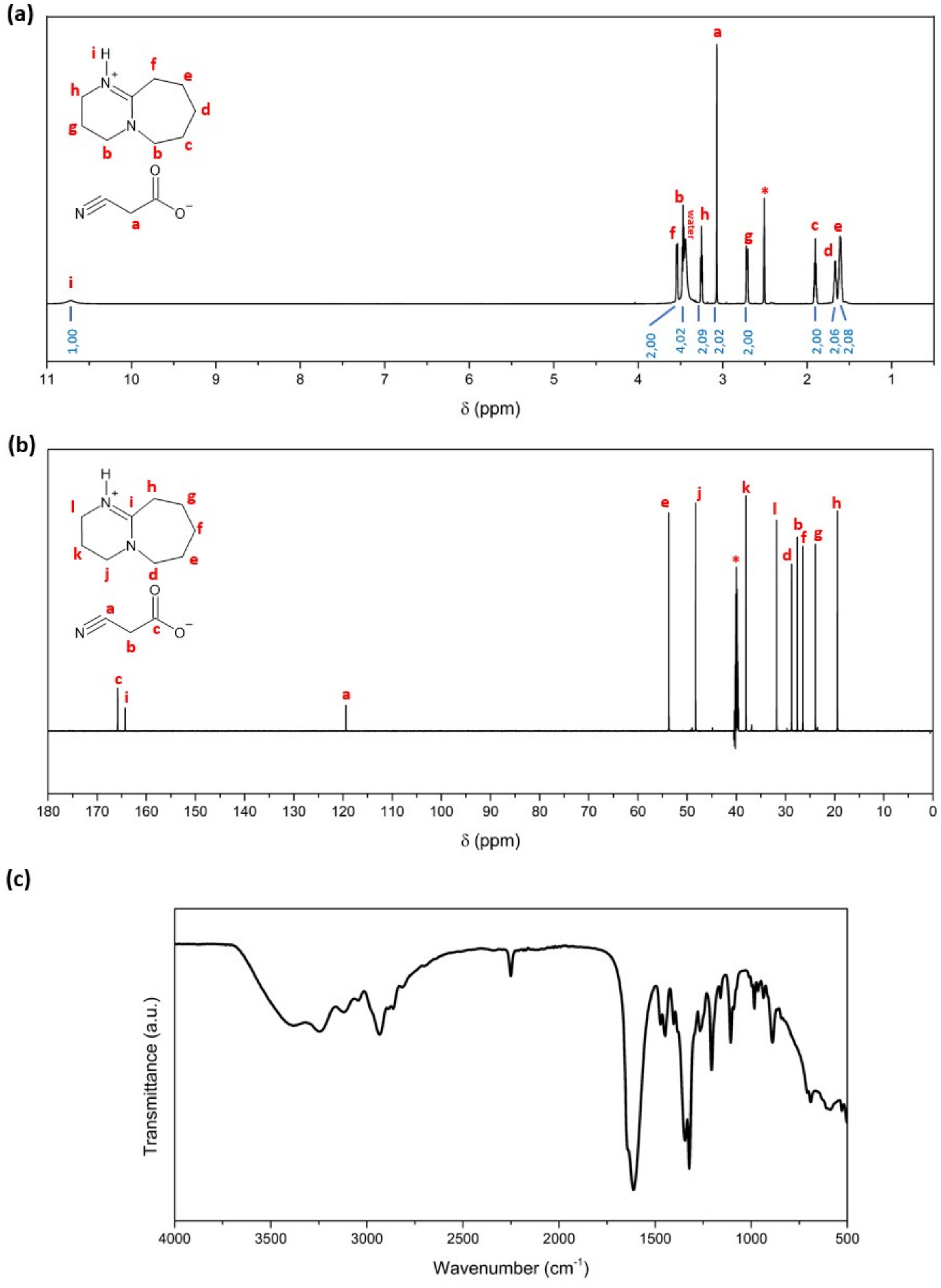

Figure S2: (a) ^{1}H NMR, (b)^{13}C NMR (600 MHz, DMSO -d_6), and (c) FTIR spectra of **1-b**.

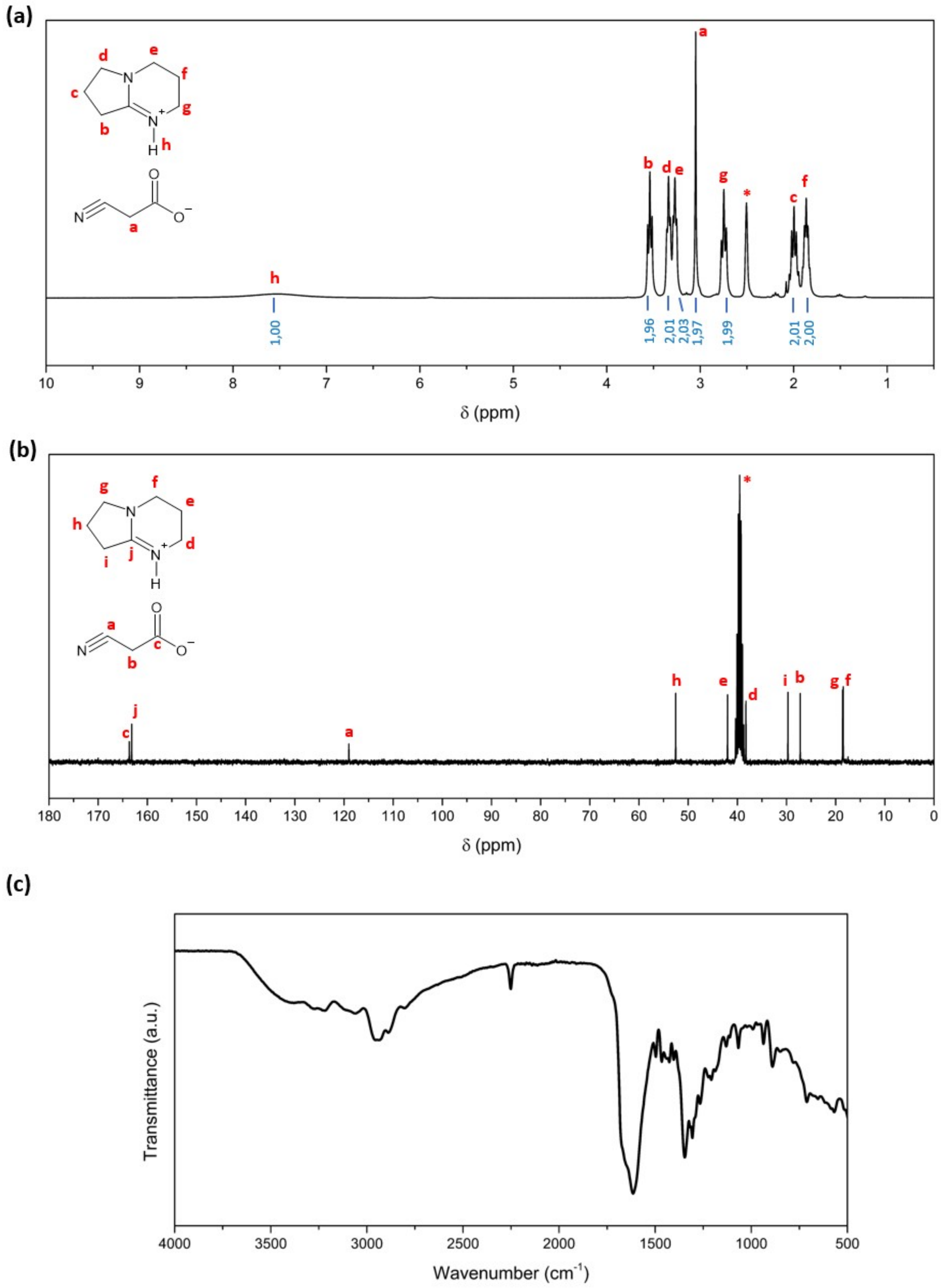

Figure S3: (a) ^{1}H NMR, (b)^{13}C NMR (600 MHz, DMSO -d_6), and (c) FTIR spectra of **1-c**.

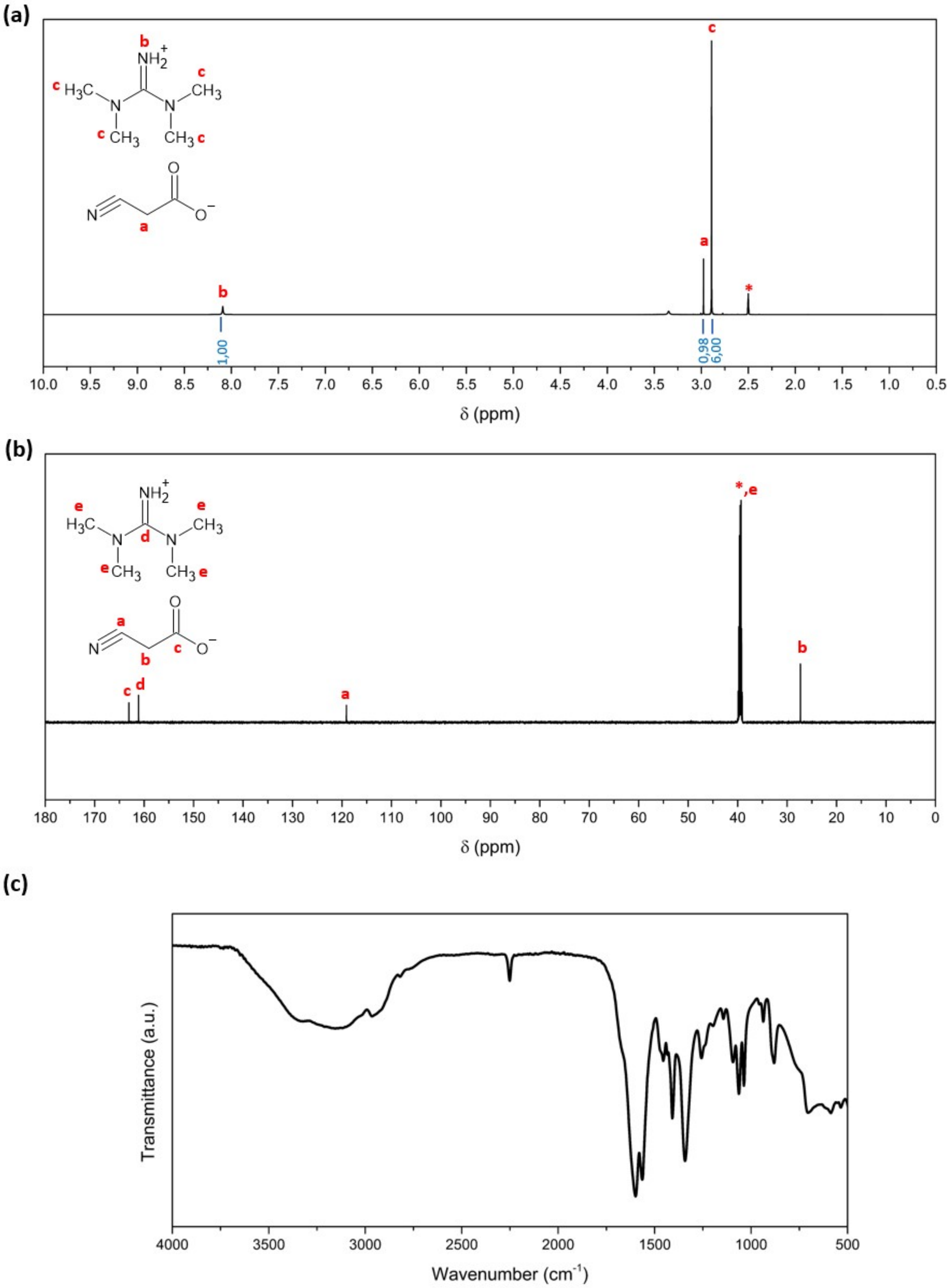

Figure S4: (a) ^{1}H NMR, (b)^{13}C NMR (600 MHz, DMSO -d_6), and (c) FTIR spectra of **1-d**.

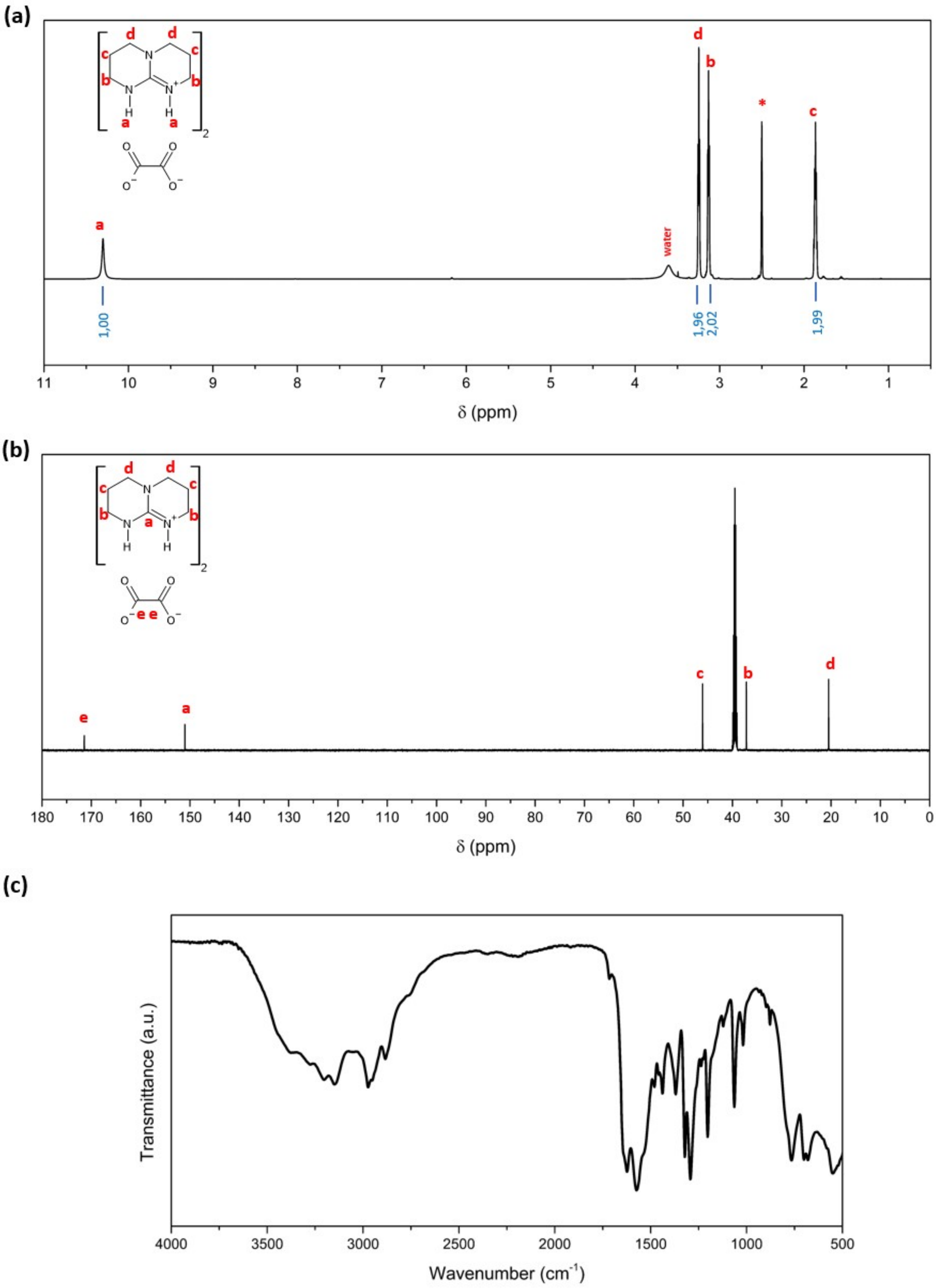

Figure S5: (a) ^{1}H NMR, (b)^{13}C NMR (600 MHz, DMSO -d_6), and (c) FTIR spectra of **2-a**.

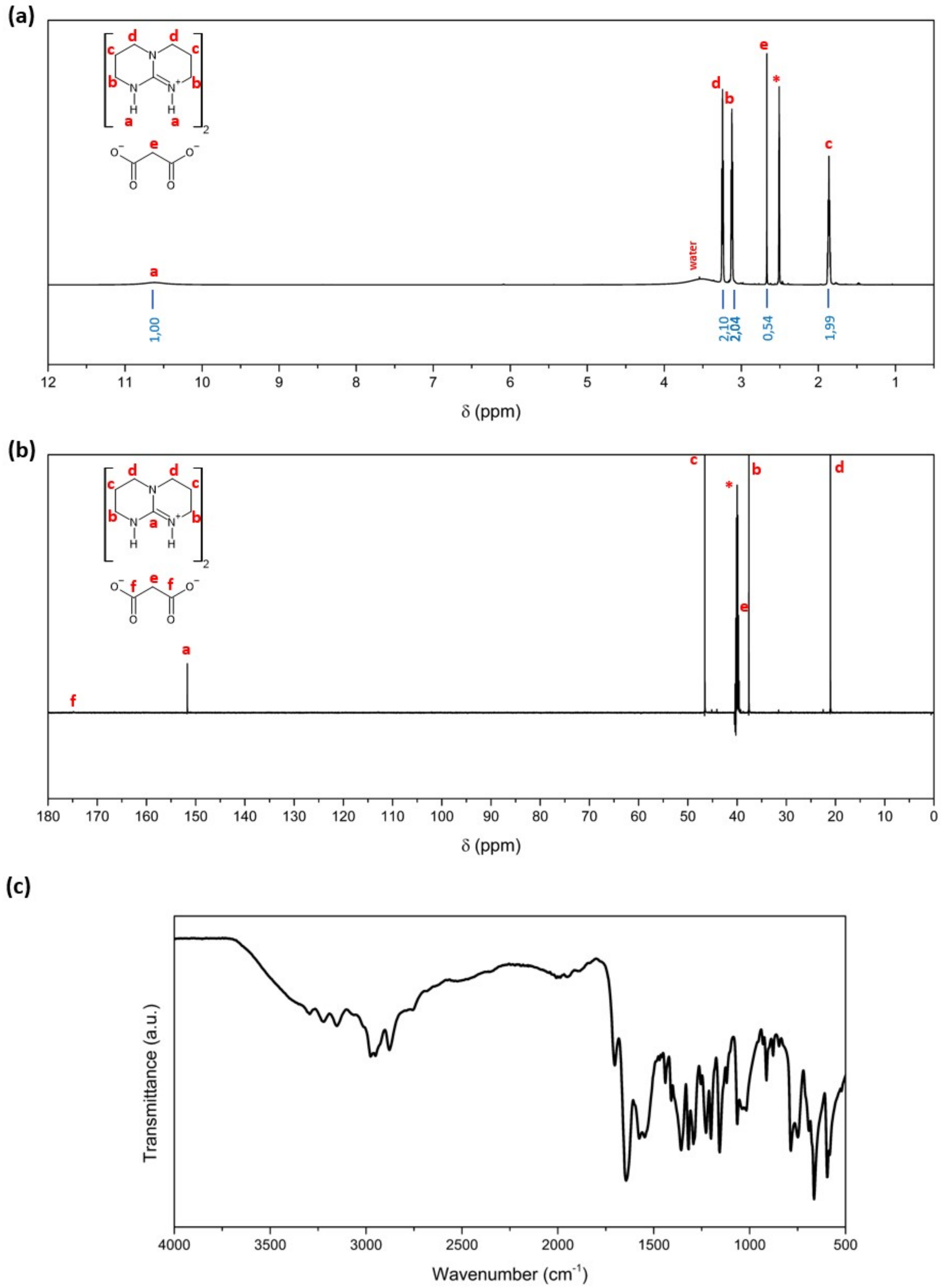

Figure S6: (a) ^{1}H NMR, (b)^{13}C NMR (600 MHz, DMSO -d$_6$), and (c) FTIR spectra of **2-b**.

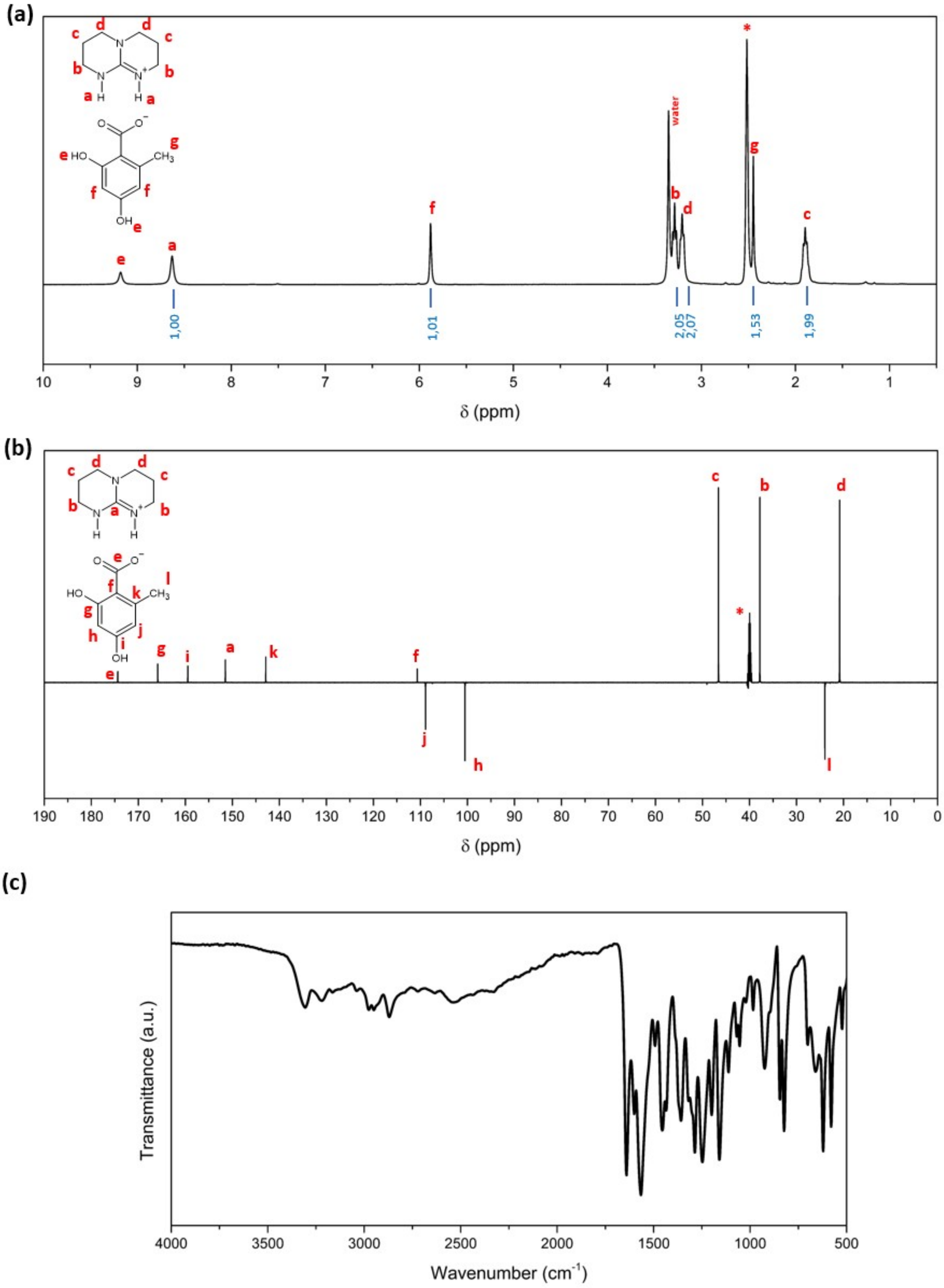

Figure S7: (a) ^{1}H NMR, (b)^{13}C NMR (600 MHz, DMSO -d_6), and (c) FTIR spectra of **2-c**.

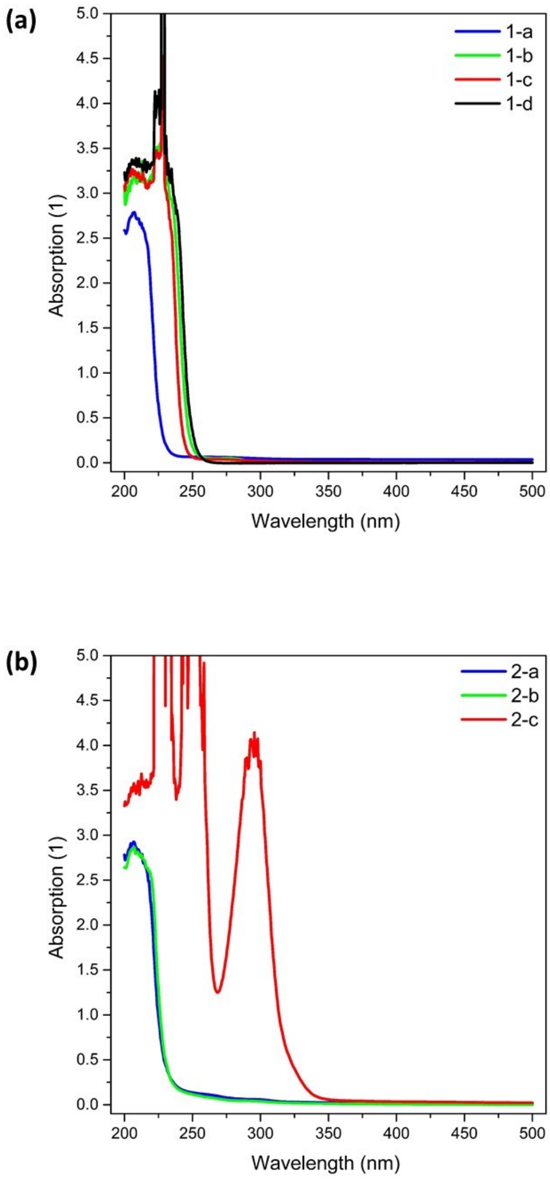

Figure S8: UV-VIS spectra of (a) **1-a-d** and(b) **2-a**-c

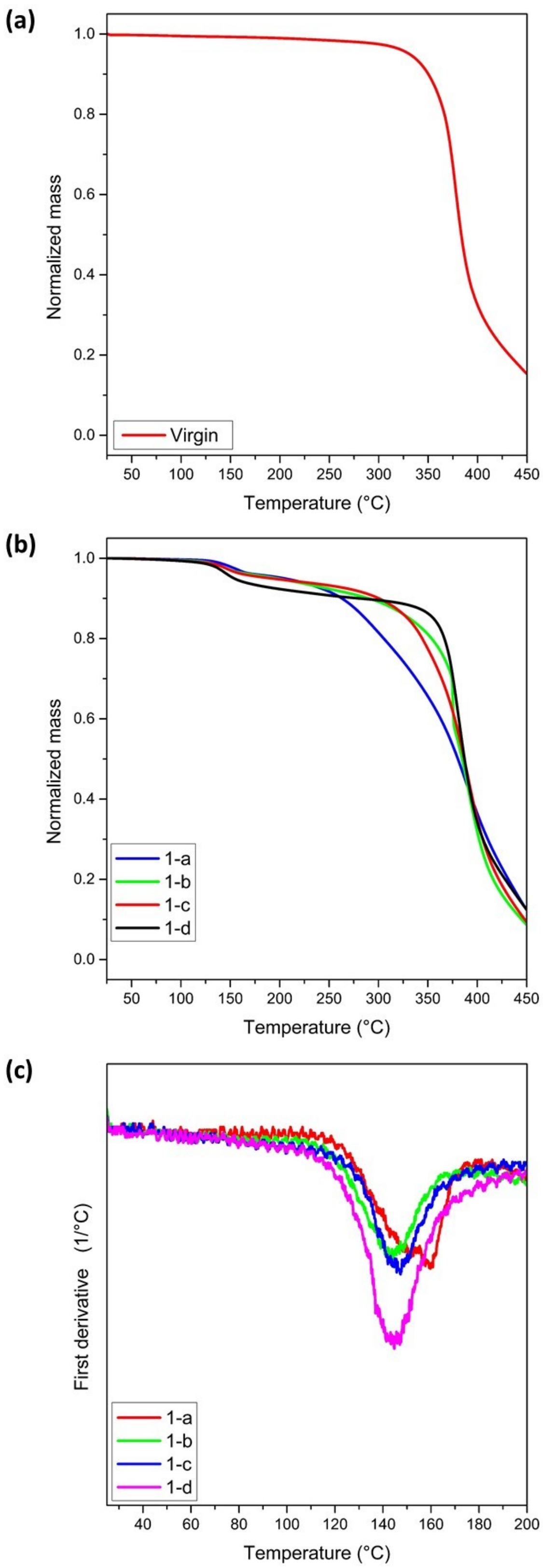

Figure S9: (a) TGA curves of the virgin thiol-ene sample without TBG (b) TGA curves of thiol-ene samples containing 10 mol% of **1-a-d** ,and (c) First derivative of TGA curves of thiol-ene samples containing 10 mol% of **1-a-d**

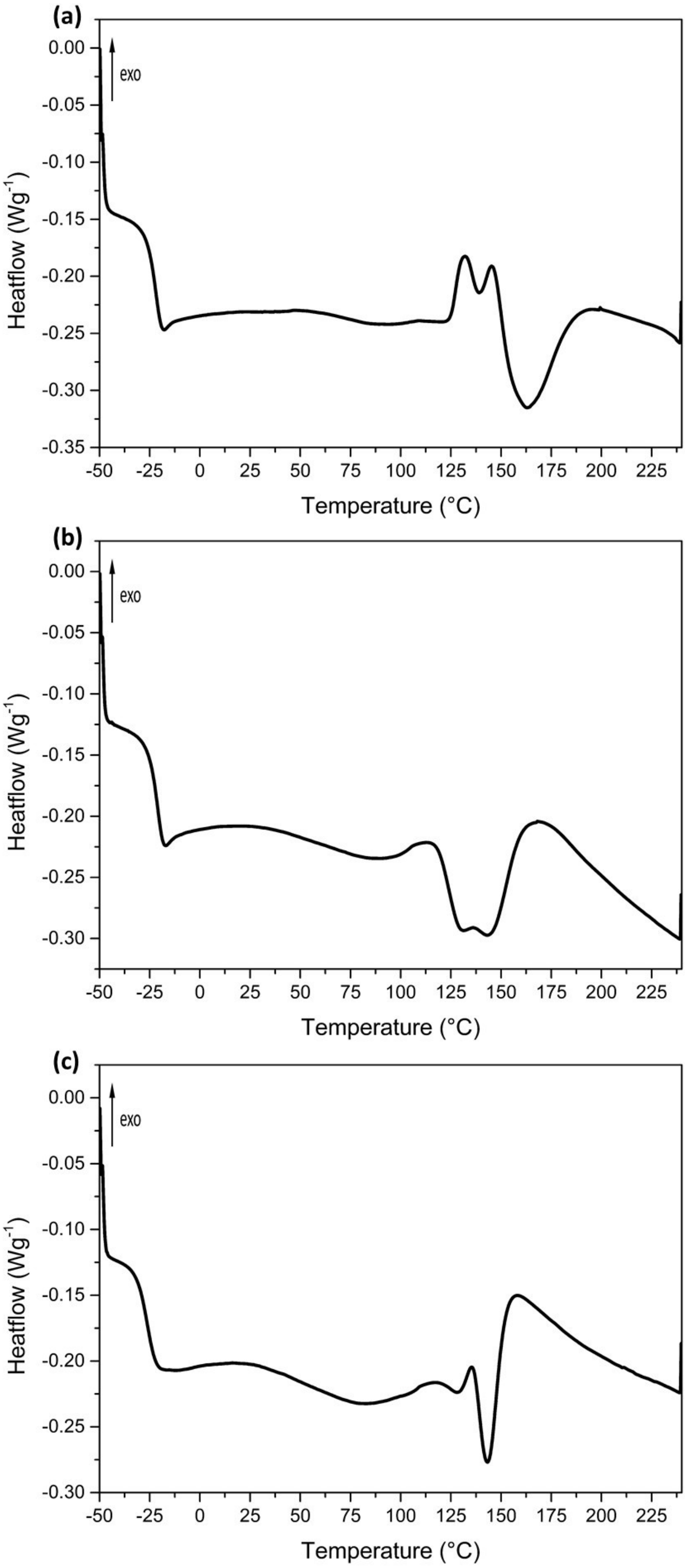

Figure S10: DSC curves of thiol-ene CANs with a heating rate of 10 Kmin^{-1}containing 10 mol% of (a) **1-a**, (b) **1-b**, and (c) **1-c**

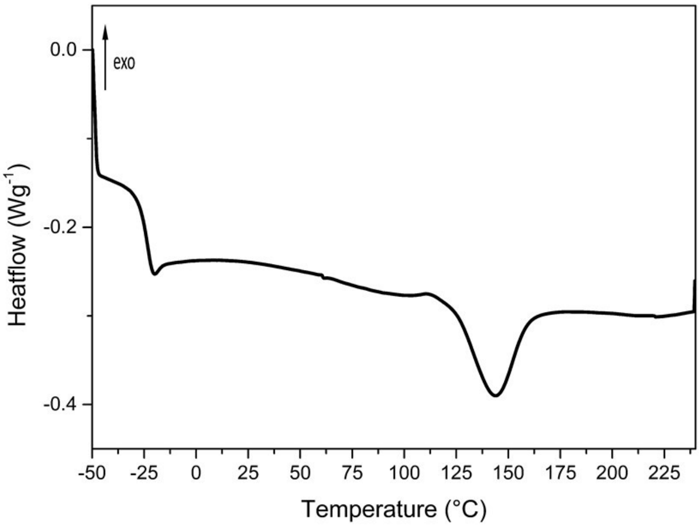

Figure S11: DSC curves of thiol-ene CANs with a heating rate of 10 Kmin^{-1}containing 10 mol% of **1-d**

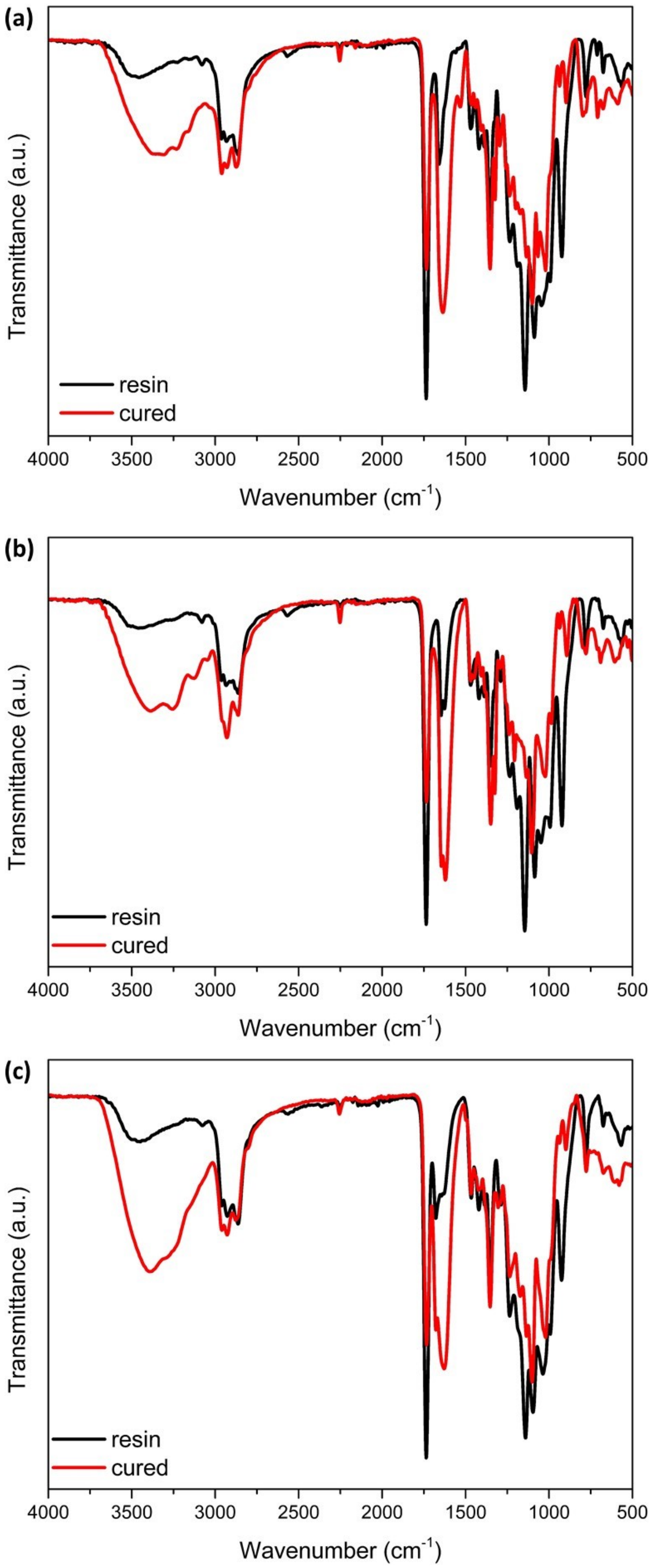

Figure S12: FTIR spectra of resin and light cured thiol-ene samples of (a) **1-a**, (b) **1-b**, and (c) **1-c**

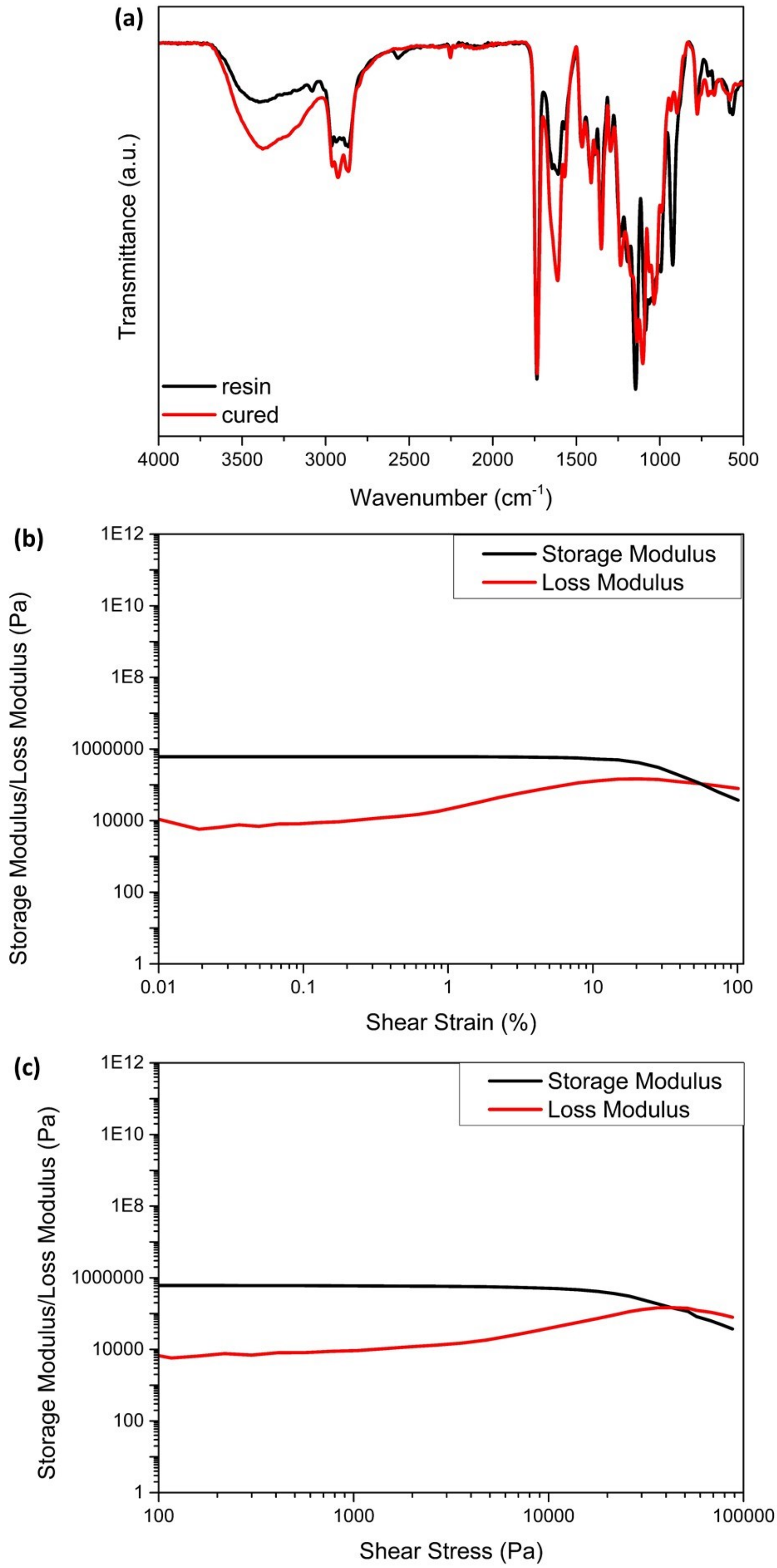

Figure S13: (a) FTIR spectra of resin and light cured thiol-ene sample **1-d**, (b) amplitude sweep – storage/loss modulus plotted against shear stress of thiol-ene sample containing **1-a**, and (c) amplitude sweep – storage/loss modulus plotted against shear strain of thiol-ene sample containing **1-a**

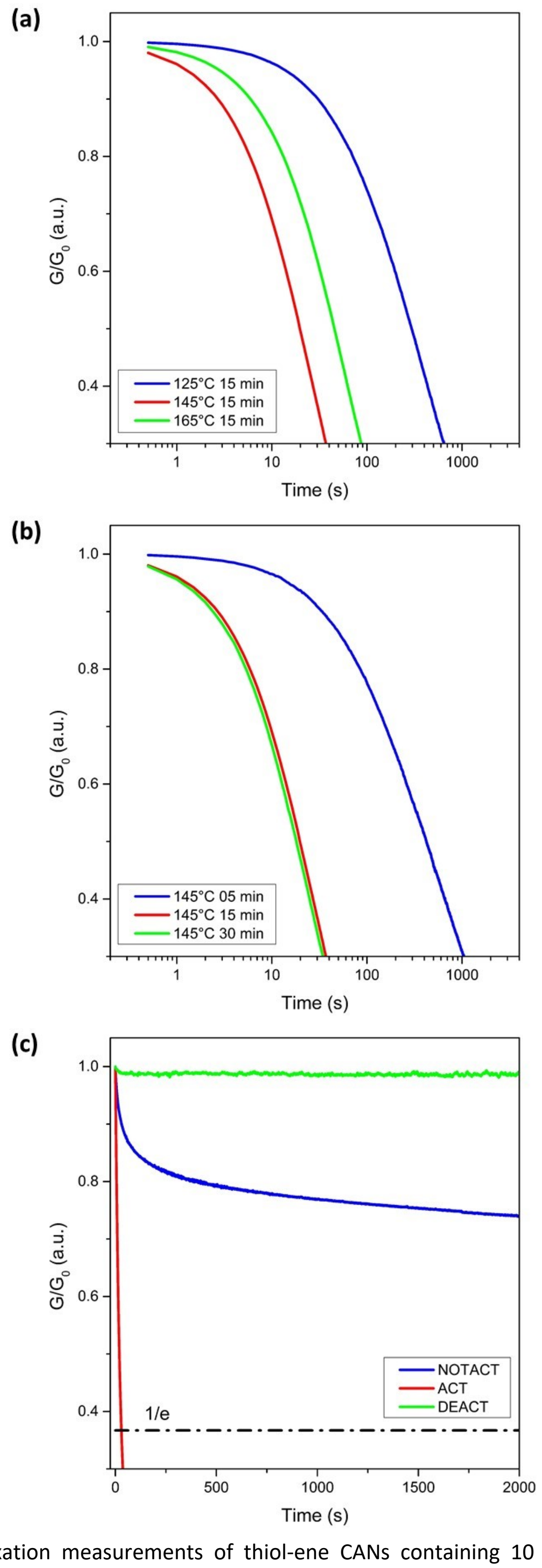

Figure S14: Stress-relaxation measurements of thiol-ene CANs containing 10 mol% **1-a** with (a) determination of the activation temperature with varying activation temperatures (125 °C; 145 °C; 165 °C) but constant activation time (15 min) and (b) determination of the activation time with varying activation times (5 min; 15 min, 30 min) but constant activation temperature (145°C), (c) Curves of not activated, activated and deactivated samples.

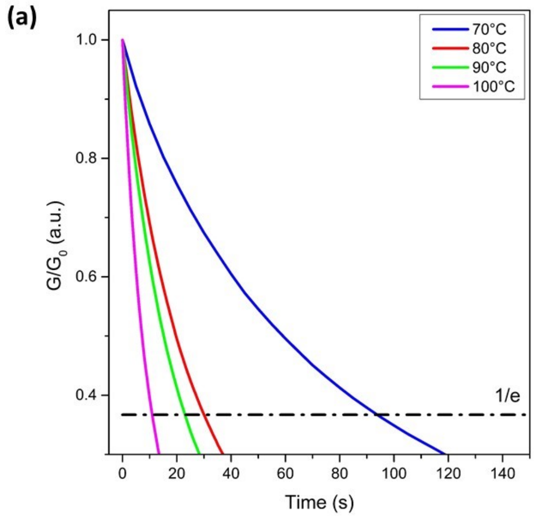

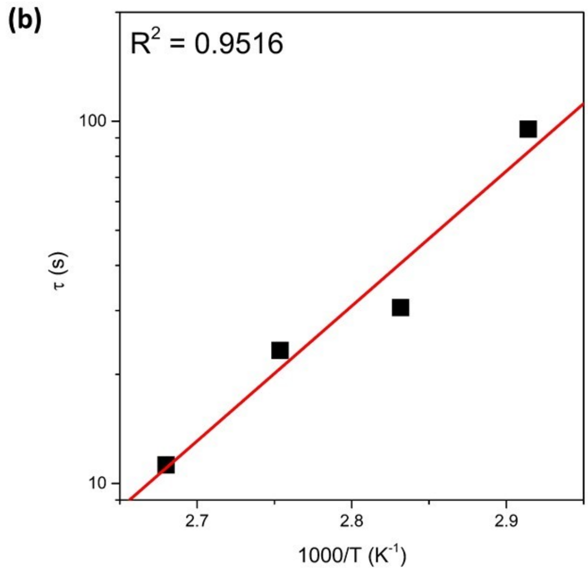

Figure S15: Stress-relaxation measurements of thiol–ene CANs containing 10 mol% **1-a** with (a) temperature-dependent stress relaxation, determined stepwise in 10 °C increments between 70 and 100 °C. (b) Temperature dependence of the characteristic relaxation times (τ*) extracted from Kohlrausch–Williams–Watts (KWW) fits.

Table S3: Characteristic relaxation times (τ*) obtained from KWW fitting of stress-relaxation curves for thiol–ene CANs containing 10 mol% **1-a** at different measurement temperatures after thermal activation.

**Temperature**	**1000/T**	**τ***
**°C**	**K^{-1}**	**s**
70	2.91	96
80	2.83	31
90	2.75	23
100	2.68	11

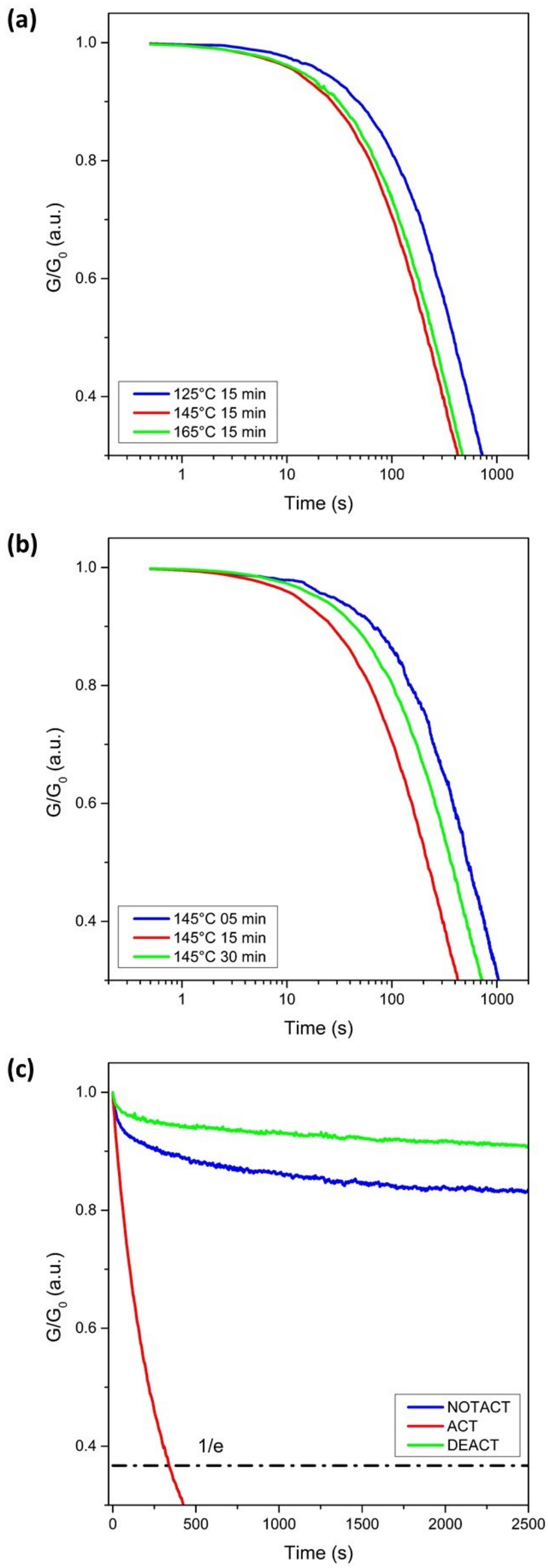

Figure S16: Stress-relaxation measurements of thiol-ene CANs containing 10 mol% **1-b** with (a) determination of the activation temperature with varying activation temperatures (125 °C; 145 °C; 165 °C) but constant activation time (15 min) and (b) determination of the activation time with varying activation times (5 min; 15 min, 30 min) but constant activation temperature (145 °C), (c) Curves of not activated, activated and deactivated samples.

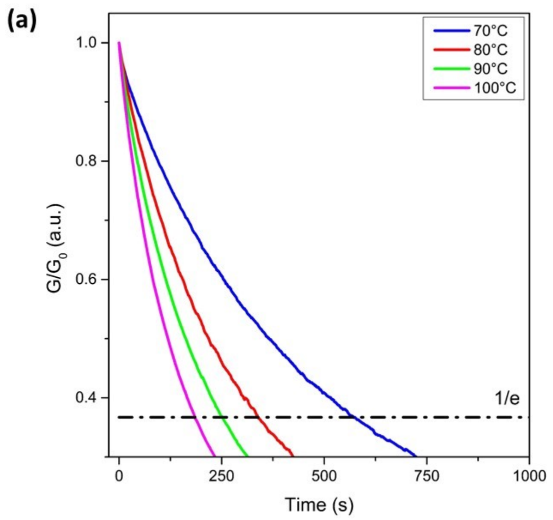

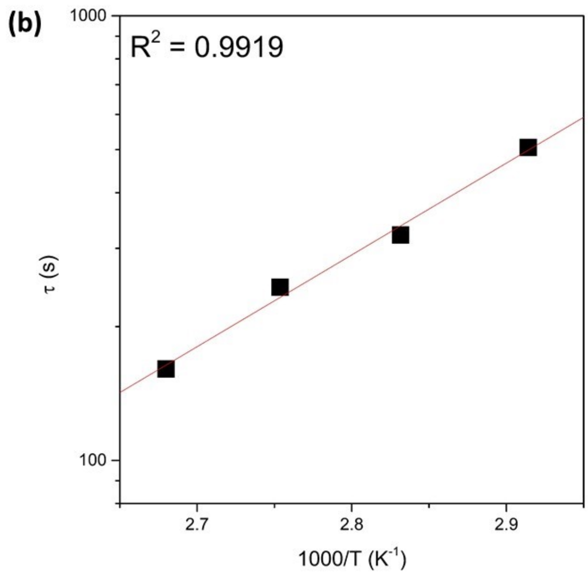

Figure S17 Stress-relaxation measurements of thiol–ene CANs containing 10 mol% **1-b** with (a) temperature-dependent stress relaxation, determined stepwise in 10 °C increments between 70 and 100 °C. (b) Temperature dependence of the characteristic relaxation times (τ*) extracted from Kohlrausch–Williams–Watts (KWW) fits.

Table S4: Characteristic relaxation times (τ*) obtained from KWW fitting of stress-relaxation curves for thiol–ene CANs containing 10 mol% **1-b** at different measurement temperatures after thermal activation.

**Temperature**	**1000/T**	**τ***
**°C**	**K^{-1}**	**s**
70	2.91	506
80	2.83	321
90	2.75	245
100	2.68	161

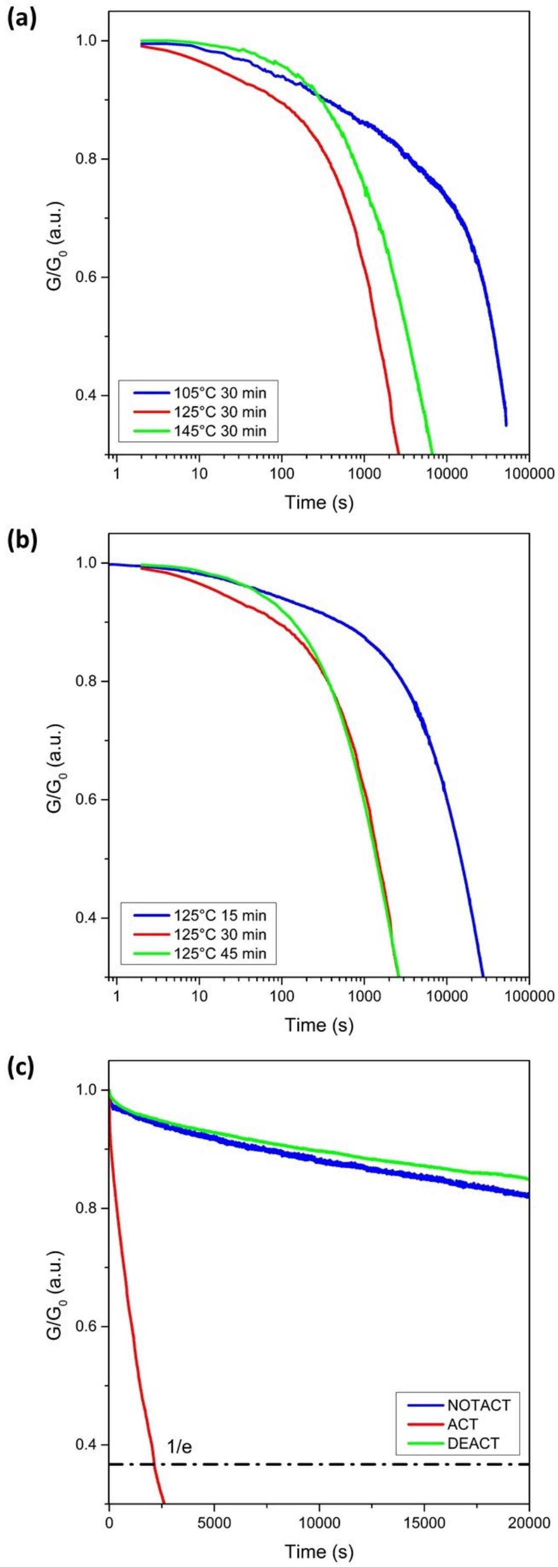

Figure S18: Stress-relaxation measurements of thiol-ene CANs containing 10 mol% **1-c** with (a) determination of the activation temperature with varying activation temperatures (125 °C; 145 °C; 165 °C) but constant activation time (15 min) and (b) determination of the activation time with varying activation times (5 min; 15 min, 30 min) but constant activation temperature (145 °C), (c) Curves of not activated, activated and deactivated samples.

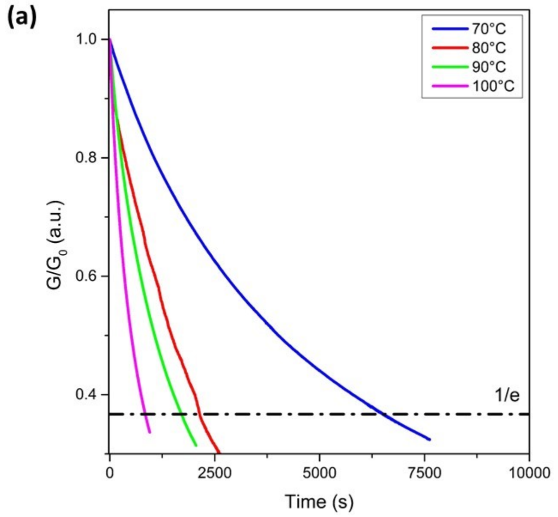

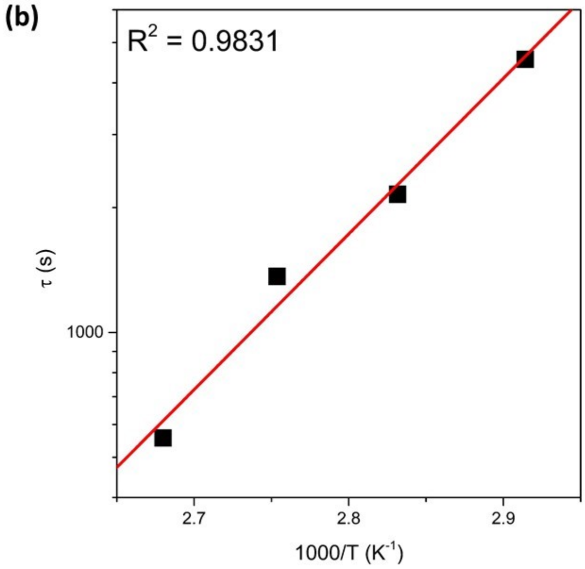

Figure S19: Stress-relaxation measurements of thiol–ene CANs containing 10 mol% **1-c** with (a) temperature-dependent stress relaxation, determined stepwise in 10 °C increments between 70 and 100 °C. (b) Temperature dependence of the characteristic relaxation times (τ*) extracted from Kohlrausch–Williams–Watts (KWW) fits.

Table S5: Characteristic relaxation times (τ*) obtained from KWW fitting of stress-relaxation curves for thiol–ene CANs containing 10 mol% **1-c** at different measurement temperatures after thermal activation.

**Temperature**	**1000/T**	**τ***
**°C**	**K^{-1}**	**s**
70	2.91	4554
80	2.83	2154
90	2.75	1365
100	2.68	557

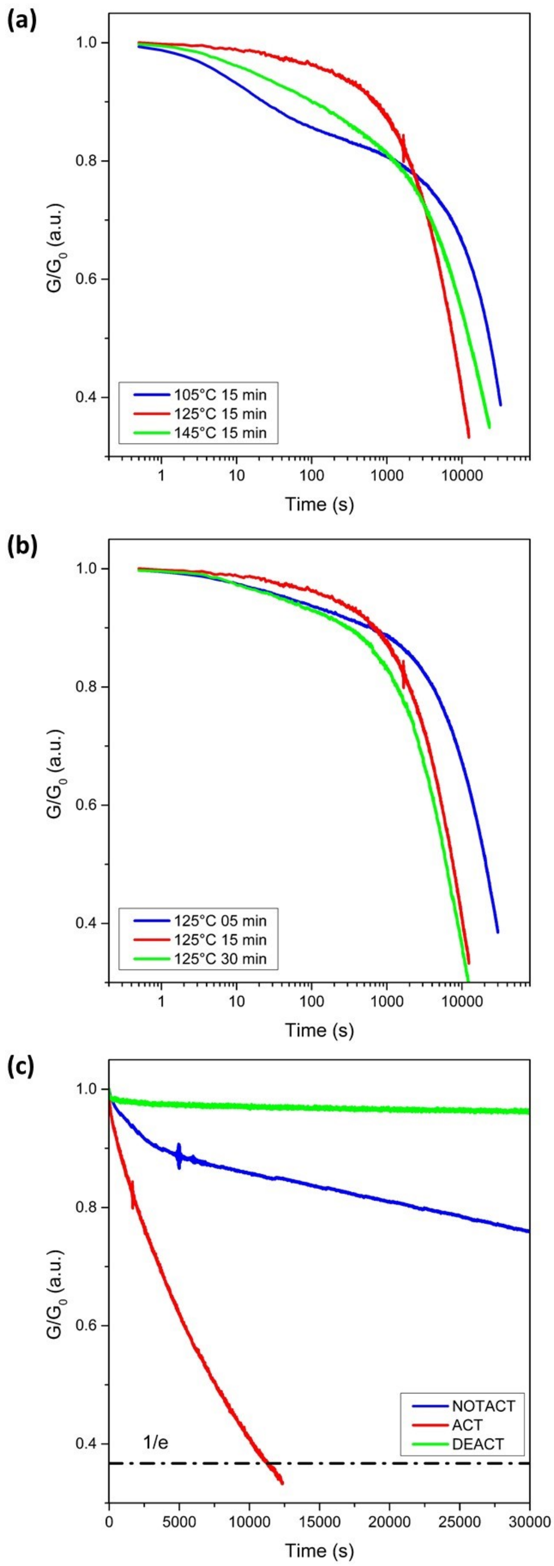

Figure S20: Stress-relaxation measurements of thiol-ene CANs containing 10 mol% **1-d** with (a) determination of the activation temperature with varying activation temperatures (125 °C; 145 °C; 165 °C) but constant activation time (15 min) and (b) determination of the activation time with varying activation times (5 min; 15 min, 30 min) but constant activation temperature (145 °C), (c) Curves of not activated, activated and deactivated samples.

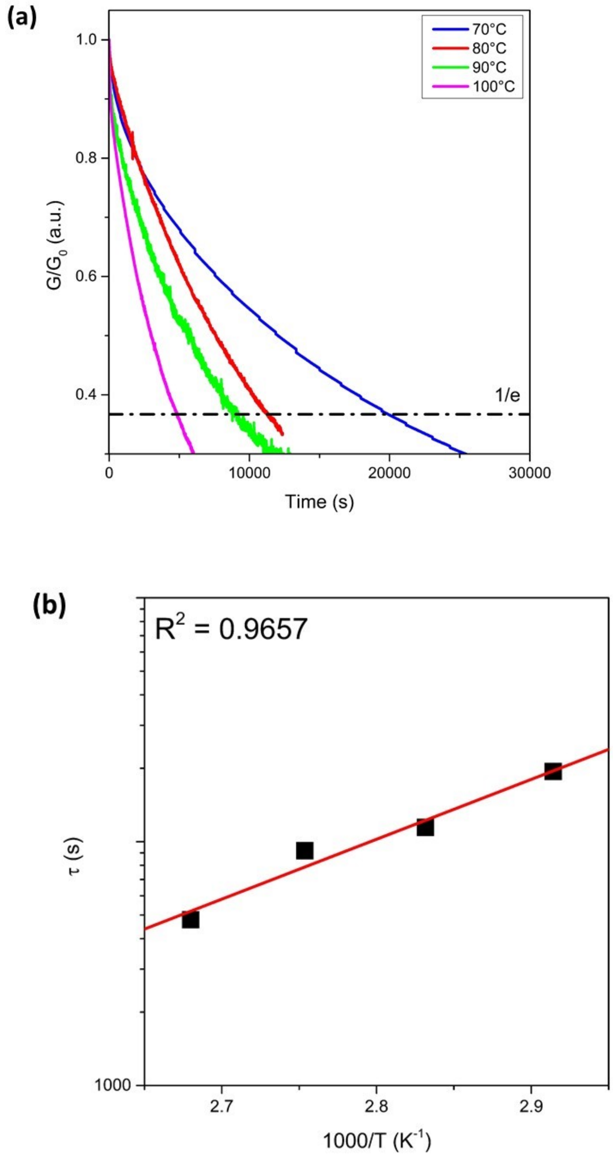

Figure S21: Stress-relaxation measurements of thiol–ene CANs containing 10 mol% **1-d** with (a) temperature-dependent stress relaxation, determined stepwise in 10 °C increments between 70 and 100 °C. (b) Temperature dependence of the characteristic relaxation times (τ*) extracted from Kohlrausch–Williams–Watts (KWW) fits.

Table S6: Characteristic relaxation times (τ*) obtained from KWW fitting of stress-relaxation curves for thiol–ene CANs containing 10 mol% **1-d** at different measurement temperatures after thermal activation.

**Temperature**	**1000/T**	**τ***
**°C**	**K^{-1}**	**s**
70	2.91	19409
80	2.83	11448
90	2.75	9176
100	2.68	4778

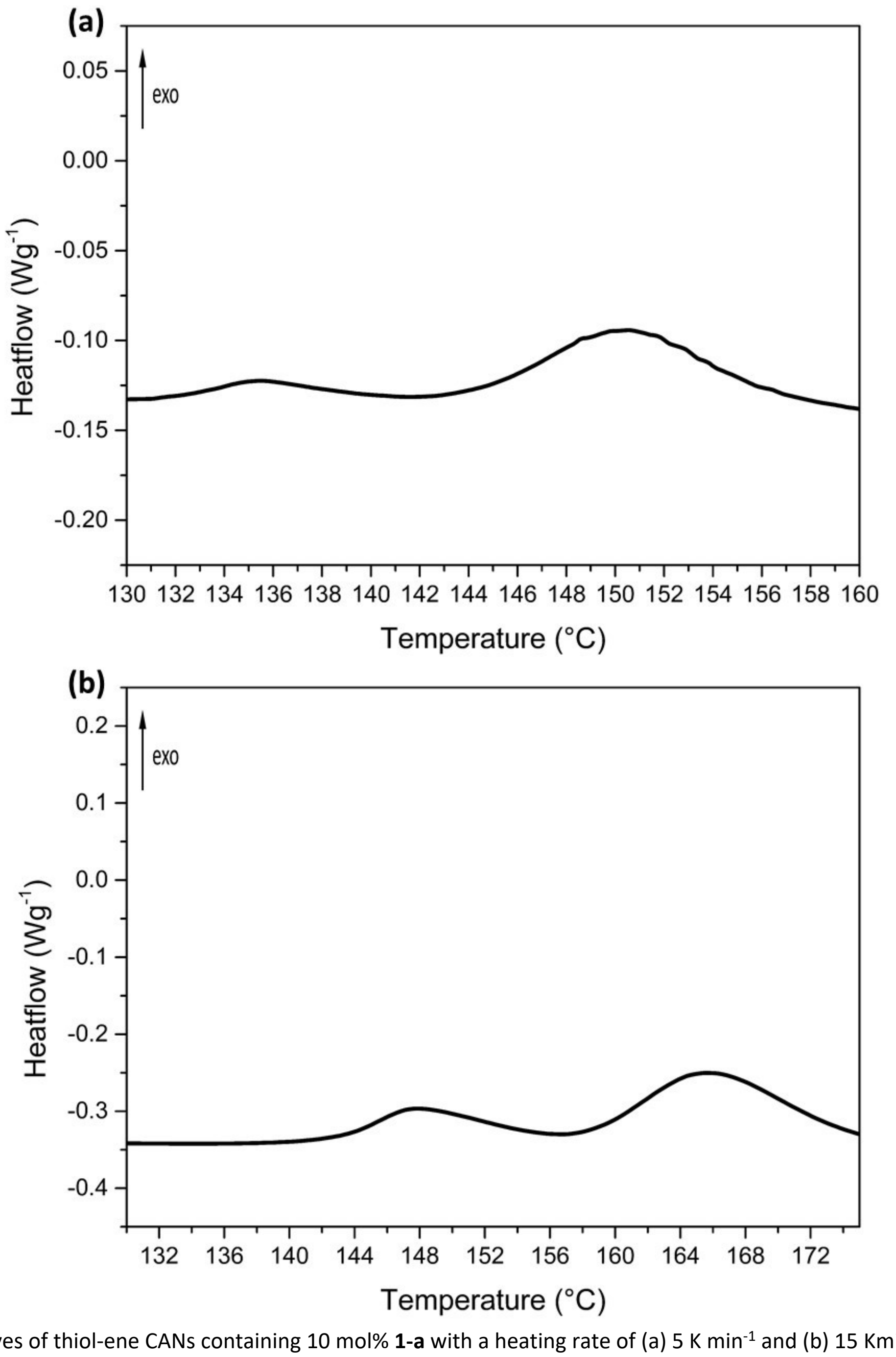

Figure S22: DSC curves of thiol-ene CANs containing 10 mol% **1-a** with a heating rate of (a) 5 K min^{-1} and (b) 15 $Kmin^{-1}$

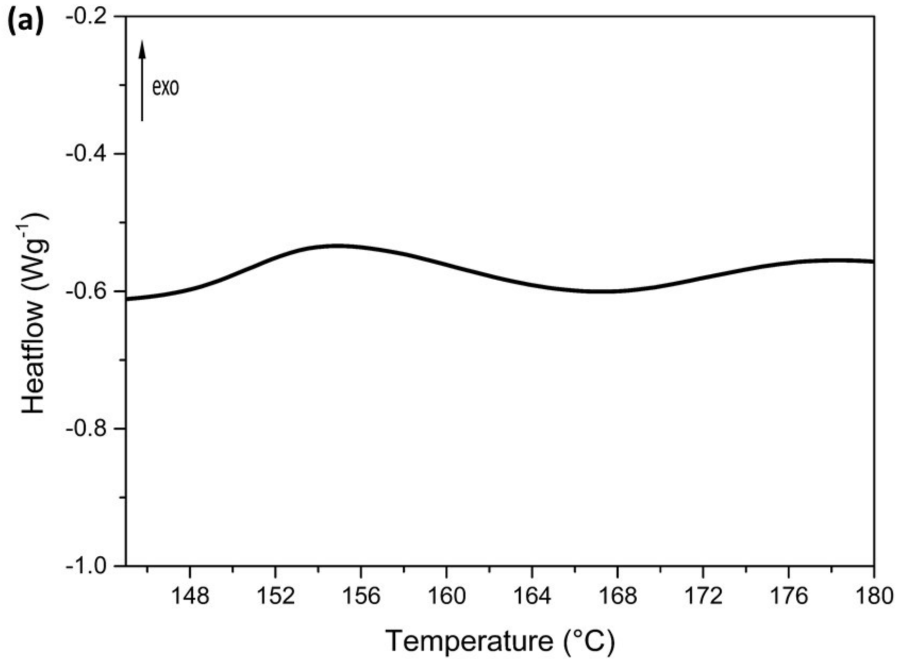

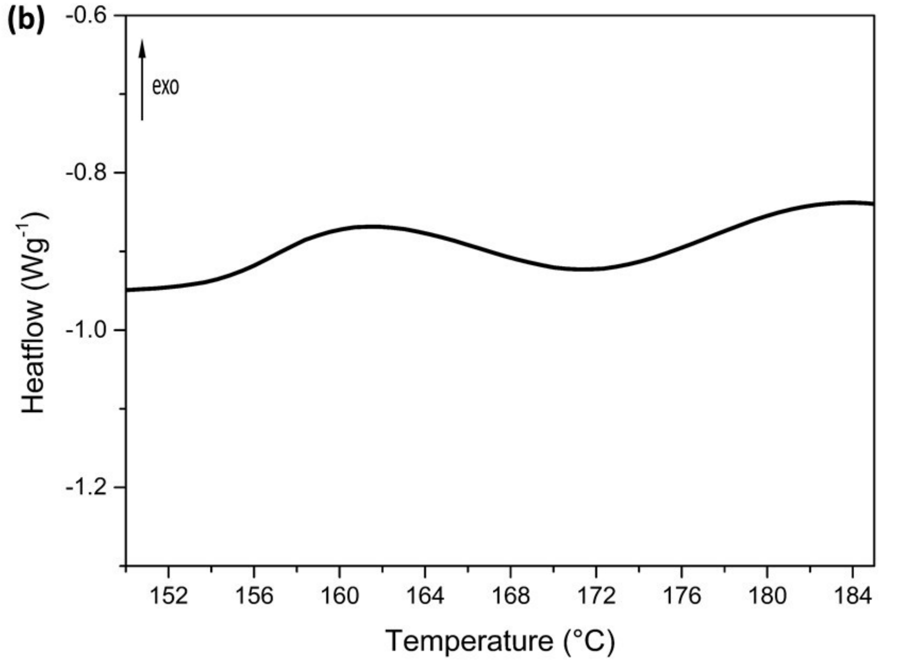

Figure S23: DSC curves of thiol-ene CANs containing 10 mol% **1-a** with a heating rate of (a) 25 K min^{-1} and (b) 35 $Kmin^{-1}$

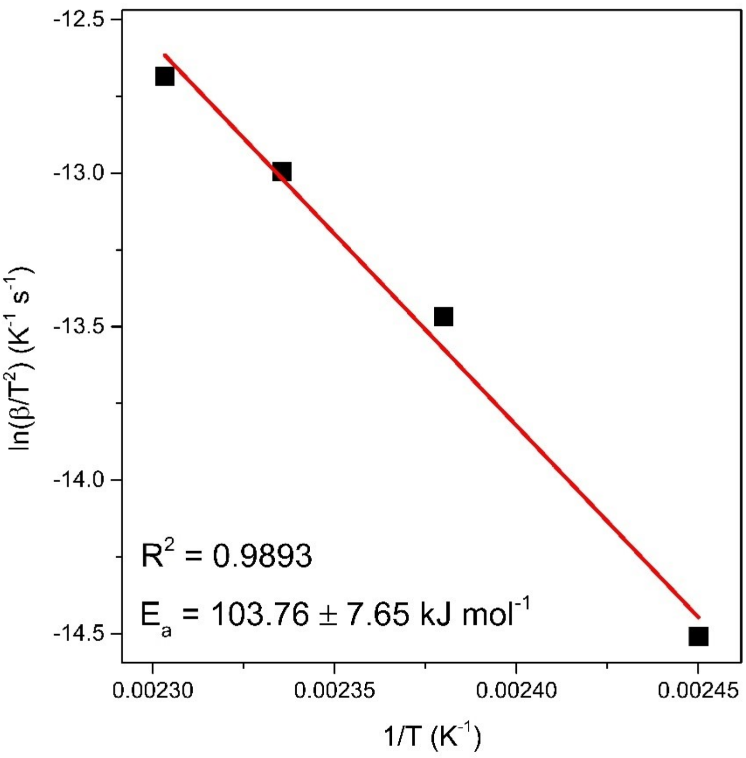

±

Figure S24: Linear regression of ln(β/T^2) versus 1/T for **1-a** in a thiol-ene CAN. The slope of the regression line was used to calculate the apparent activation energy according to the Kissinger equation, yielding E_a =103.76± 7.65 kJ mol^{-1}.

Table S7: Required data for the Kissinger analysis for the heating rates 5, 15, 25 and 35 K min^{-1}.

**Heating rate**	**T_{ACT}**	**Heating rate**	**T_{ACT}**	**1/T**	**1/T**	**T^2**	**ln(b/T^2)**
K min^{-1}	°C	K s^{-1}	K	1/K	1000/K	K^2	
5	135	0.0833	408.15	0.002450	2.45	166586	-14.51
15	147	0.2500	420.15	0.002380	2.38	176526	-13.47
25	155	0.4167	428.15	0.002336	2.34	183312	-12.99
35	161	0.5833	434.15	0.002303	2.30	188486	-12.69

(a)

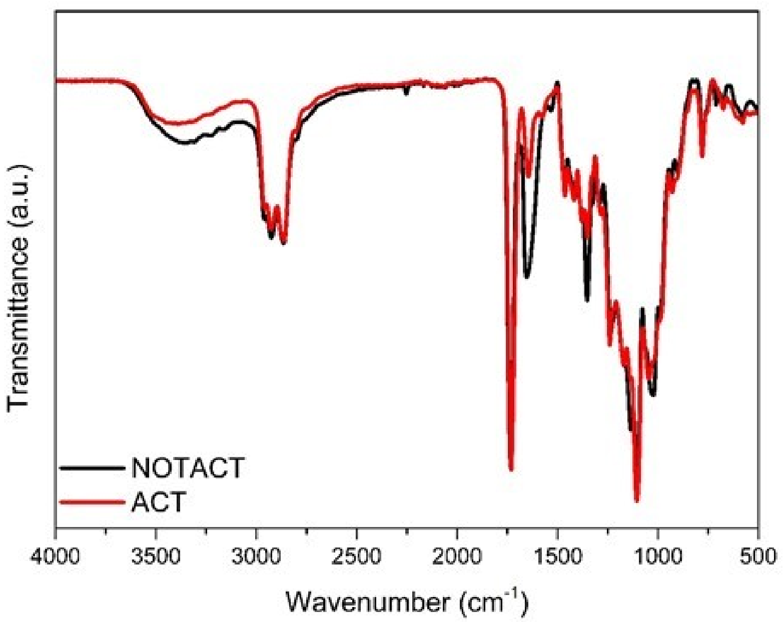

(b)

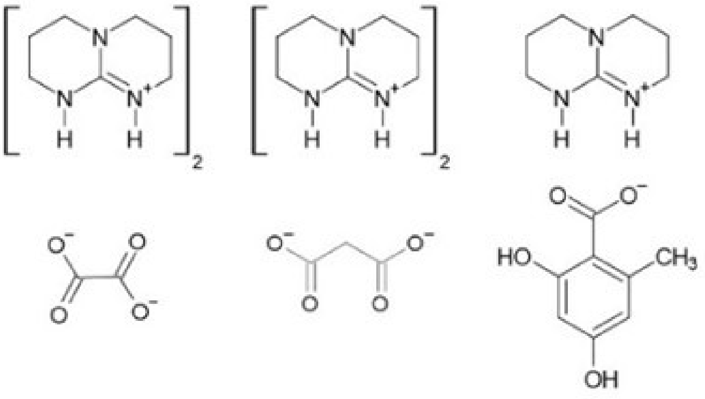

2-a **2-b** **2-c**

TBG		2-a	2-b	2-c
$T_{pH\text{-}ACT}$	°C	200	200	160
ΔpH		2.65	4.47	2.26

Figure S25: (a) FTIR spectra of cured thiol-ene sample containing 10 mol% of **1-a** not thermally activated (NOTACT) and thermally treated for 15 min at 145°C (t_{ACT} and T_{ACT}) in inert nitrogen atmosphere (ACT) (b) Chemical structures and thermal behaviour of TBD-based thermo base generators (TBGs **2-a-c**) with varying anion structure. The activation temperatures in DMSO/water solution ($T_{pH\text{-}ACT}$) and corresponding ΔpH values upon thermal activation are listed.

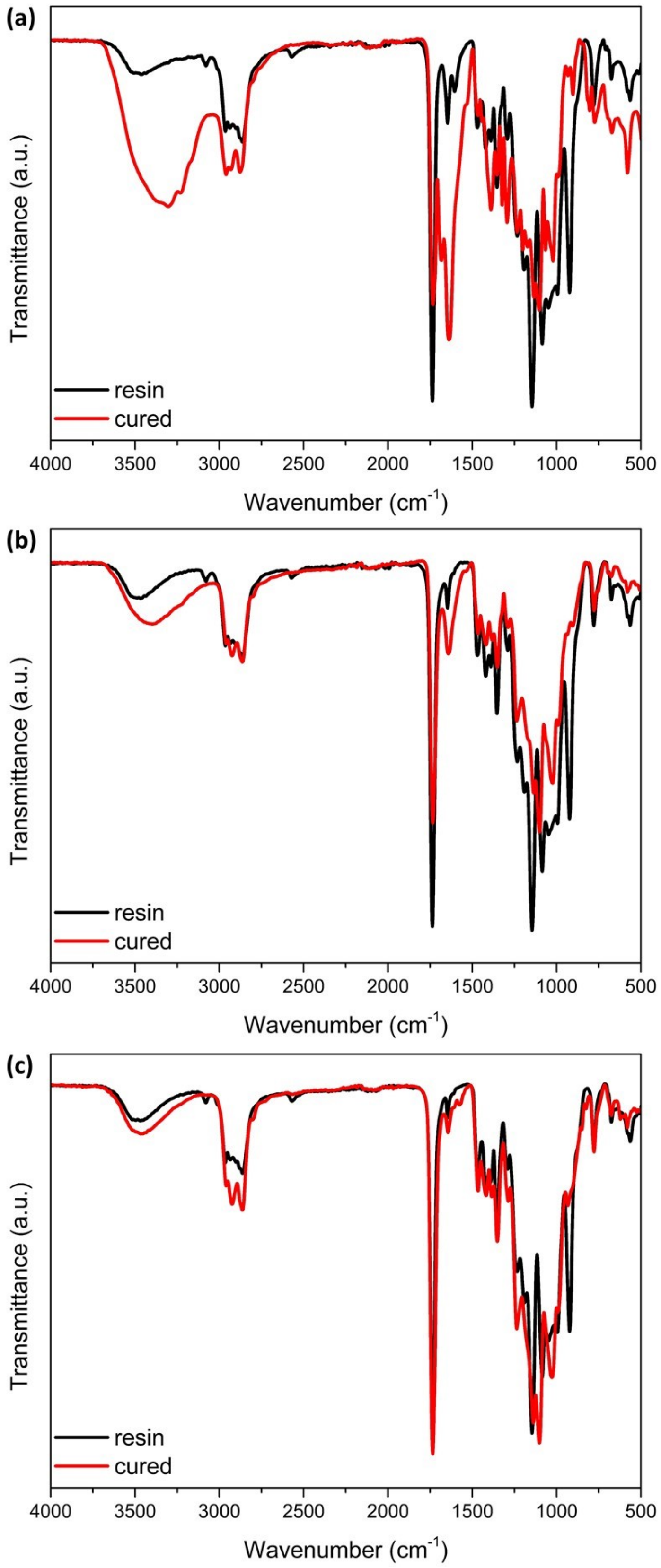

Figure S26: FTIR spectra of resin and light cured thiol-ene samples of (a) **2-a** (b) **2-b** and (c) **2-c**

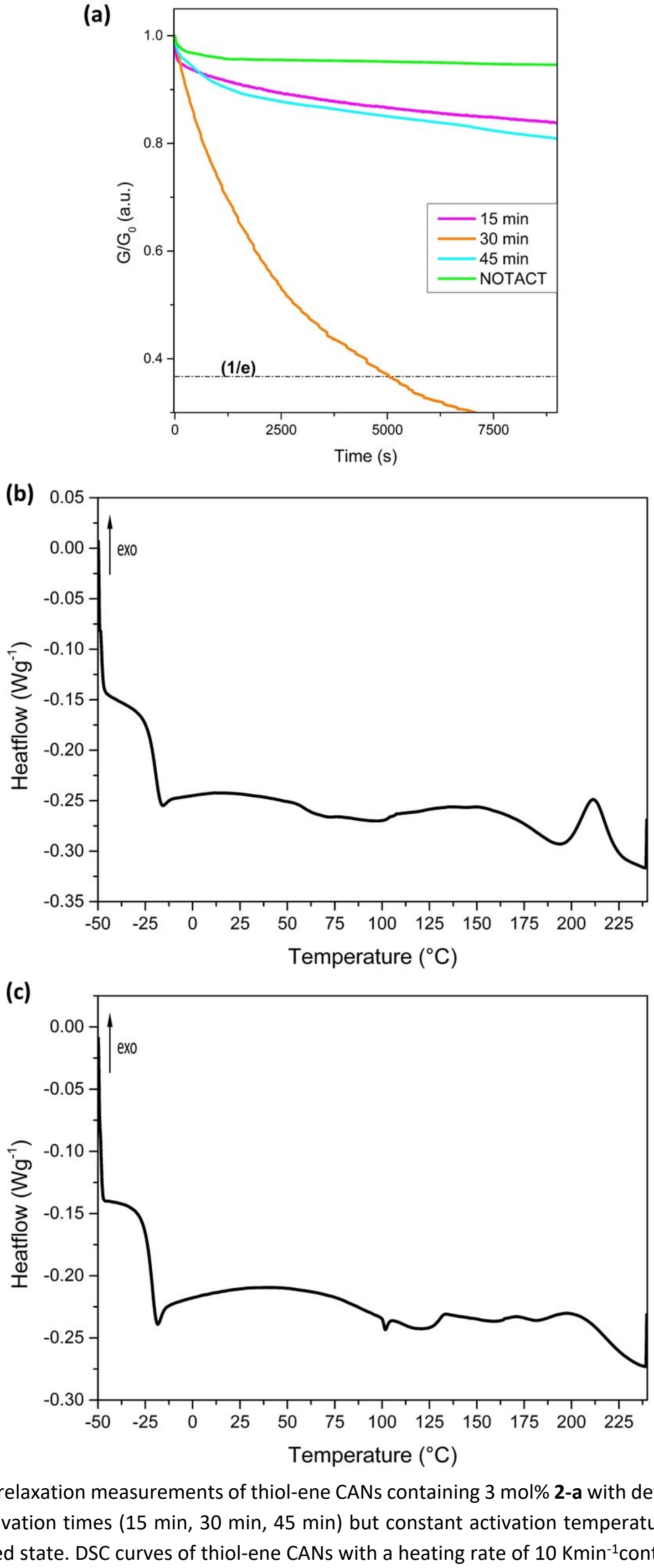

Figure S27: (a) Stress-relaxation measurements of thiol-ene CANs containing 3 mol% **2-a** with determination of the activation time with varying activation times (15 min, 30 min, 45 min) but constant activation temperature (190°C) compared with a sample in not-activated state. DSC curves of thiol-ene CANs with a heating rate of 10 $Kmin^{-1}$containing 3 mol% of (b) **2-a** and (c) **2-b**.

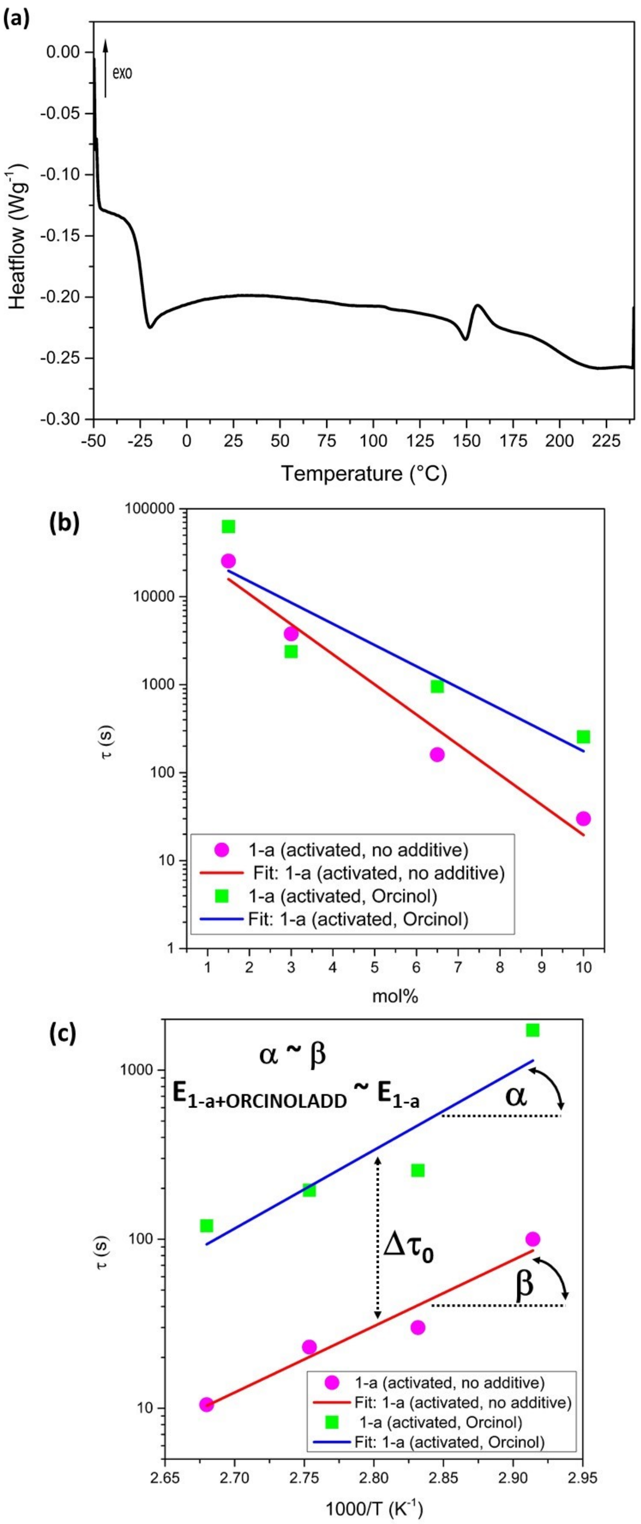

Figure S28: (a) DSC curve of thiol-ene CANs with a heating rate of 10 $Kmin^{-1}$containing 3 mol% of **2-c**. (b) Required relaxation times to reach the normalized stress-relaxation value for the thiol–ene CANs at 80 °C with 1.5, 3.0, 6.5 and 10.0 mol% concentration of **1-a** and with and without the equal amount of orcinol as additive and prior thermal activation treatment at 145°C for 15 min. (c) Required relaxation times to reach the normalized stress-relaxation value for the thiol–ene CANs at 70, 80, 90 and 100 °C of measurement temperature with a concentration of 10 mol% of **1-a** and with and without the equal amount of orcinol as additive and prior thermal activation treatment at 145 °C for 15 min.

Table S8: Required relaxation times to reach the normalized stress-relaxation value for the thiol–ene CANs at 80 °C with 1.5, 3.0, 6.5 and 10.0 mol% concentration of **1-a** and with and without the equal amount of orcinol as additive and prior thermal activation treatment at 145 °C for 15 min.

Concentration	τ*	
Mol%	s	
	1-a (activated, no orcinol)	1-a (activated, with orcinol)
1.5	25495	62785
3.0	3792	2380
6.5	160	955
10.0	30	255

Table S9: Required relaxation times to reach the normalized stress-relaxation value for the thiol–ene CANs at 70, 80, 90 and 100 °C of measurement temperature with a concentration of 10 mol% of **1-a** and with and without the equal amount of orcinol as additive and prior thermal activation treatment at 145 °C for 15 min.

Temperature	τ*	
°C	s	
	1-a (activated, no orcinol)	1-a (activated, with orcinol)
70	100	1725
80	30	255
90	23	195
100	11	120

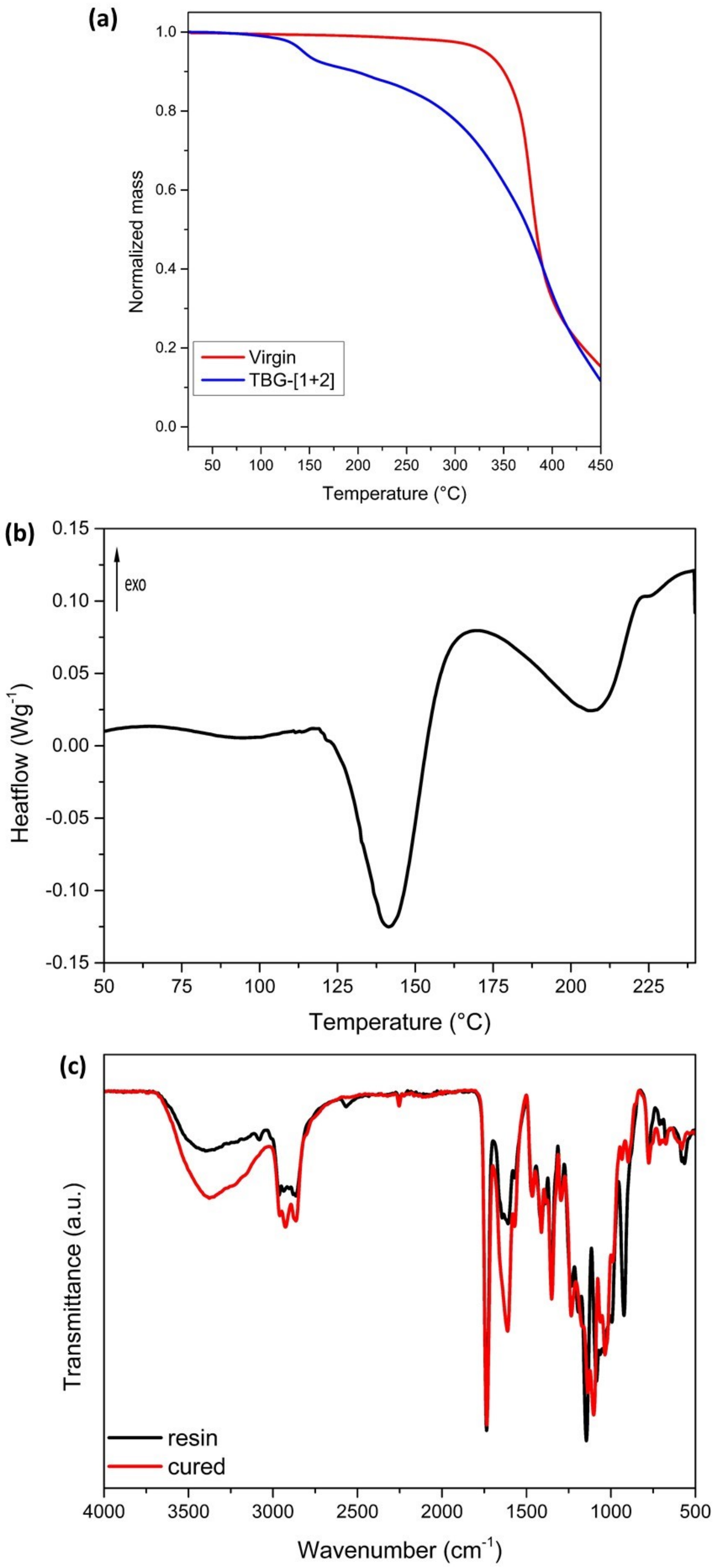

Figure S29: (a) TGA curves with a heating rate of 10 K min^{-1} of the virgin thiol-ene sample without TBG samples containing 10 mol% of **1-d** and 3 mol% of **2-a** (**TBG-[1-2]**), (b) DSC curve of thiol-ene CANs with a heating rate of 10 Kmin^{-1} containing 10 mol% of **1-d** and 3 mol% of **2-a** (**TBG-[1-2]**), and (c) FTIR spectra of resin and light cured thiol-ene samples of 10 mol% of **1-d** and 3 mol% of **2-a** (**TBG-[1-2]**).

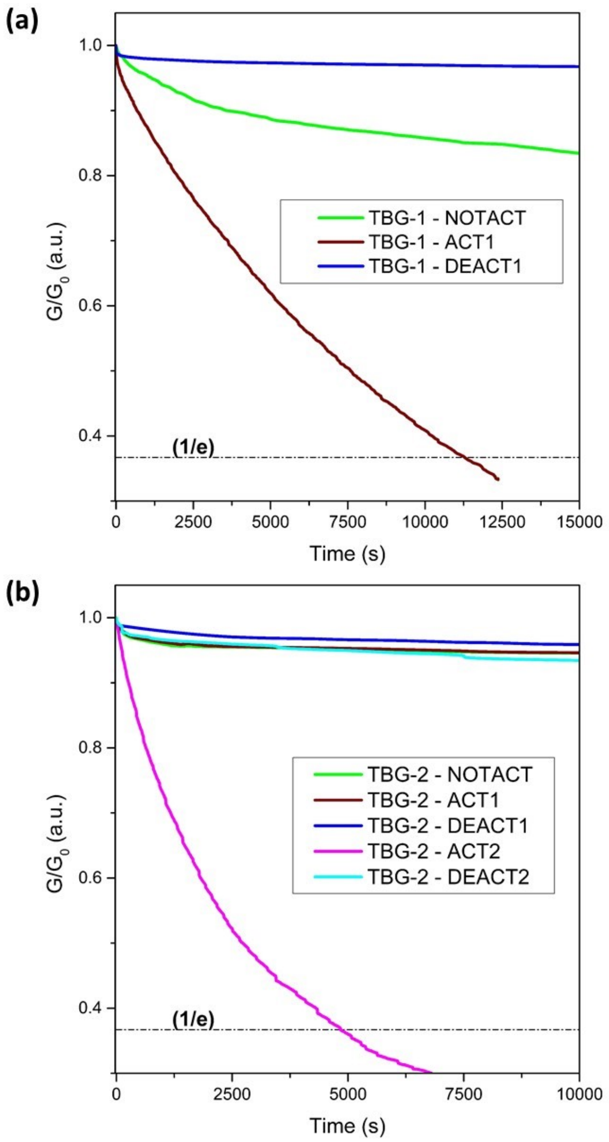

Figure S30: Stress relaxation profiles at 80 °C for different activation states of (a) **TBG-1** and (b) **TBG-2**.

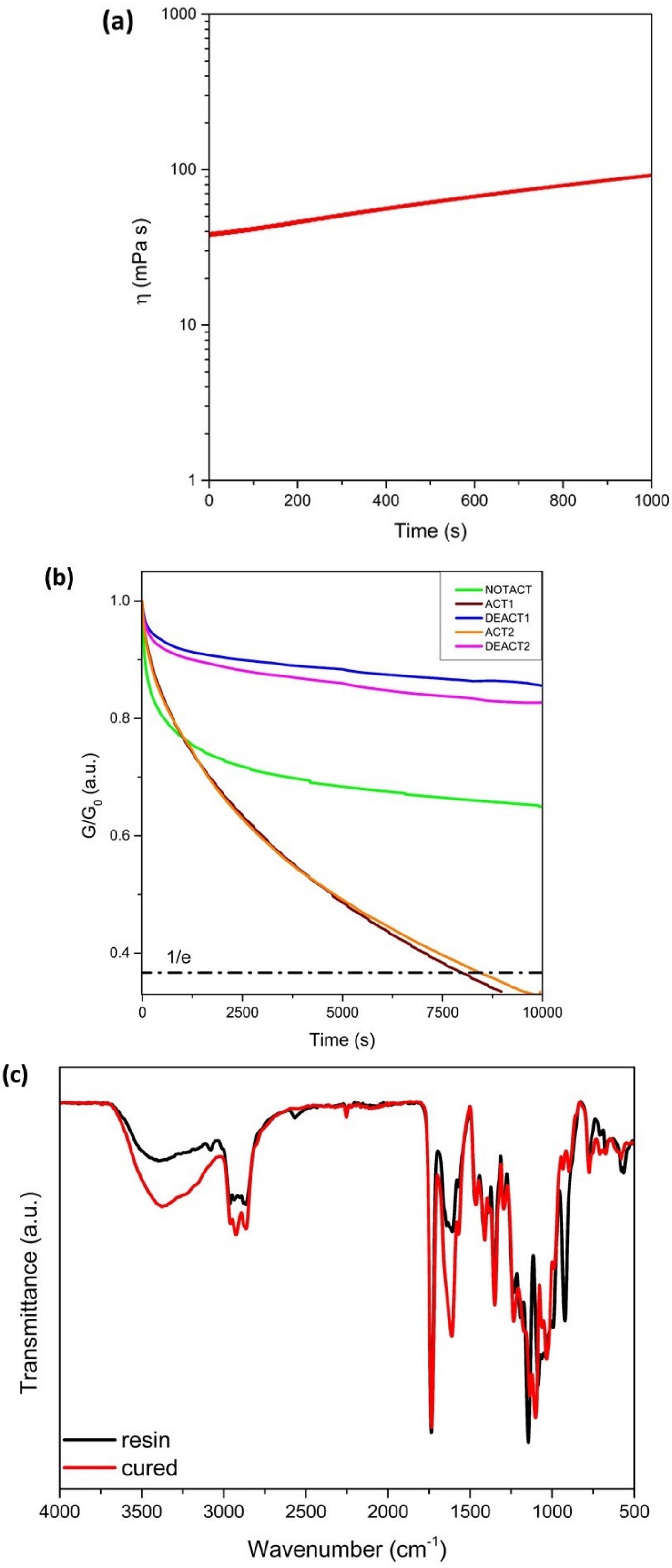

Figure S31: (a) Viscosity measurements of thiol-ene resin with 10 mol% of **1-d** and 3 mol% of **2-a** at a constant shear rate of 300 s^{-1}, (b) Stress relaxation profiles at 80 °C of additive manufactured thiol-ene samples containing 10 mol% of **1-d** and 3 mol% of **2-a** for different activation states, confirming switchable catalytic activity and full dynamic response upon dual activation, and (c) FTIR spectra of resin and 3D printed thiol-ene samples of 10 mol% of **1-d** and 3 mol% of **2-a** (**TBG-[1-2]**).

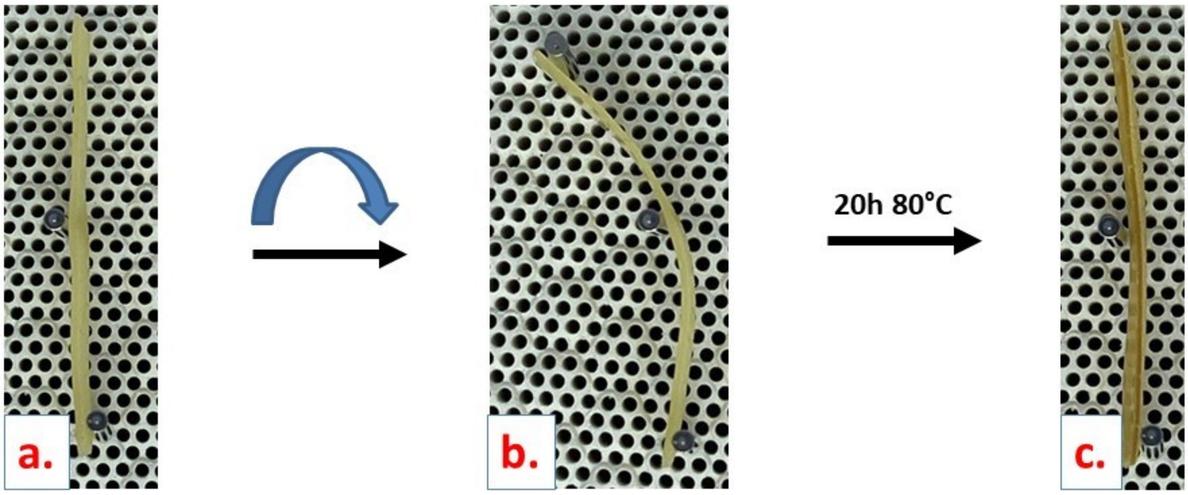

Figure S32: Reshape experiment of **TBG-[1+2]** networks in not-activated state (a) Rectangular samples of **TBG-[1+2]** in neither deformed nor thermally treated state. (b) Cold bending and mechanical fixation of sample (c) After reshape experiments at 80 °C for 20h and losing the fixation the sample retained its original bent shape.

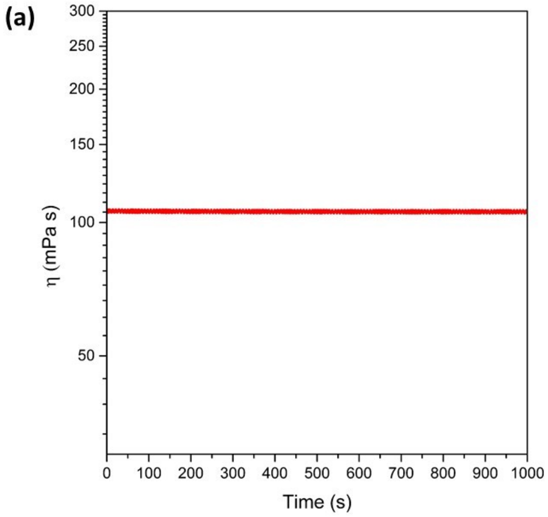

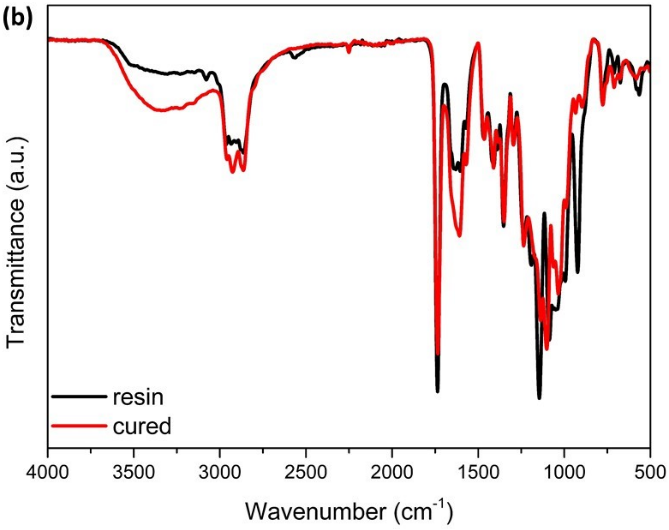

Figure S33: (a) Viscosity measurements of thiol-ene resin with 10 mol% of **1-d**, 3 mol% of **2-a** and 0.1 wt% of Sudan II at a constant shear rate of 300 s^{-1} and (b) FTIR spectra of resin and 3D printed thiol-ene samples of 10 mol% of **1-d**, 3 mol% of **2-a** (**TBG-[1-2]**) and 0.1 wt% of Sudan II.

Table S10: Retained deflection angle (Φ) from torsional deformation experiments after cooling to 40°C and unloading. Φ before unloading at 80°C after 10000 s (Φ_{10000}), Φ post unloading and settling at 40°C after 30000 s (Φ_{30000}) and reflection coefficient ($\Phi_{30000}/\Phi_{10000}$) for photocured thiol-ene samples of 10 mol% of **1-d**, 3 mol% of **2-a** (**TBG-[1-2]**) in the 5 distinct states (NOTACT, ACT1, DEACT1, ACT2, DEACT2).

State	Φ_{10000}	Φ_{30000}	$\Phi_{30000}/\Phi_{10000}$	$\Phi_{30000}/\Phi_{10000}$
-	°	°	-	%
NOTACT	51.744	4.735	0.0915	9.15
ACT1	51.744	25.304	0.4890	48.90
DEACT1	51.744	7.070	0.1366	13.66
ACT2	51.744	32.778	0.6335	63.35
DEACT2	51.744	2.713	0.0524	5.24

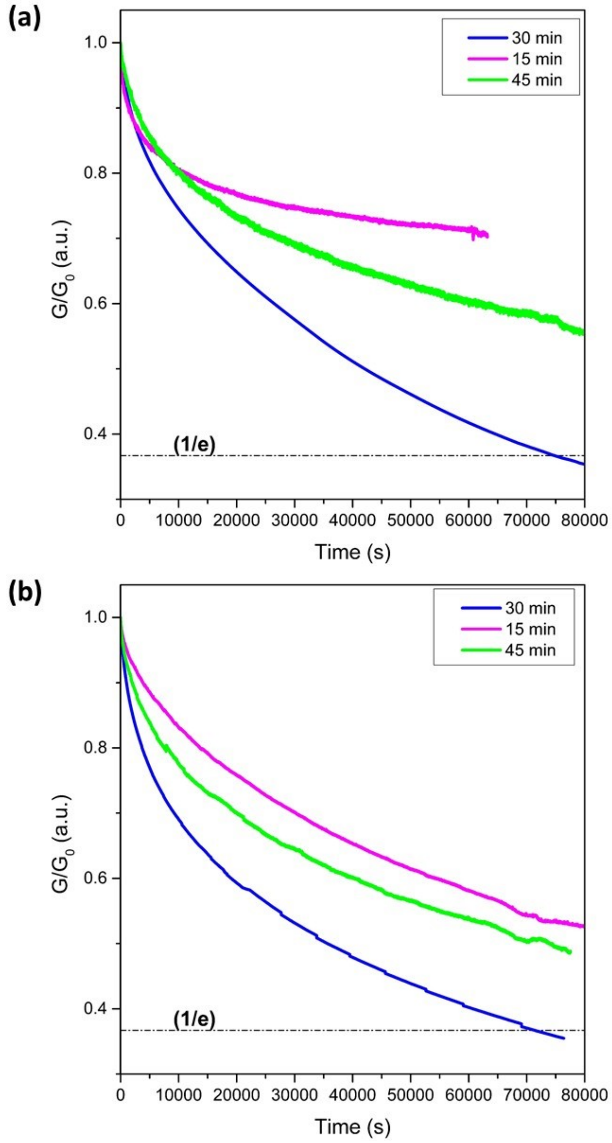

Figure S34: Stress-relaxation measurements of thiol-ene CANs containing 3 mol% of TBGs with determination of the activation time with varying activation times (15 min, 30 min, 45 min) with (a) **2-b** at a constant activation temperature of 180 °C and (b) **2-c** at a constant activation temperature of 170 °C.

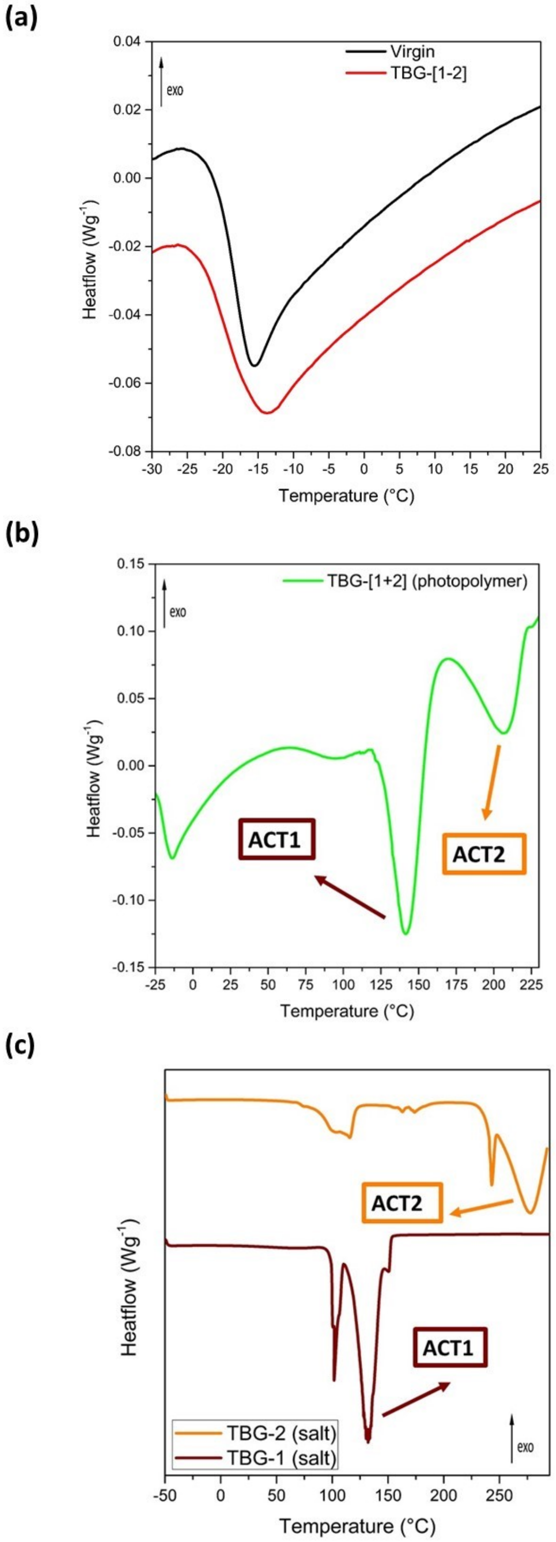

Figure S35: (a) DSC curve of thiol-ene CANs with a heating rate of 10 Kmin^{-1} containing 10 mol% of **1-d** and 3 mol% of **2-a** (**TBG-[1-2]**) and virgin sample, (b) DSC curve of thiol-ene CANs with a heating rate of 10 Kmin^{-1} containing 10 mol% of **1-d** and 3 mol% of **2-a** (**TBG-[1-2]**), (c)) DSC curve of **TBG-1** and **TBG-2** salt with a heating rate of 10 Kmin^{-1}.

References

1 a) M. U. Mayer-Kriehuber, E. Sattler, J. Krämer, D. Reisinger, D. Bautista-Anguís, J. Uher, E. Vidovic and S. Schlögl, *Monatsh Chem*, 2025; b) M. U. Mayer-Kriehuber, E. Sattler, D. Reisinger, D. Bautista-Anguís, S. Gaca, C. Schmidleitner, I. Duretek, E. Vidovic and S. Schlögl, *RSC Adv.*, 2025, **15**, 35265–35280;
